# Dectin-1 Activation Exacerbates Obesity and Insulin Resistance in the Absence of MyD88

**DOI:** 10.1016/j.celrep.2017.05.059

**Published:** 2017-06-13

**Authors:** Angela Castoldi, Vinicius Andrade-Oliveira, Cristhiane Favero Aguiar, Mariane Tami Amano, Jennifer Lee, Marcelli Terumi Miyagi, Marcela Teatin Latância, Tarcio Teodoro Braga, Marina Burgos da Silva, Aline Ignácio, Joanna Darck Carola Correia Lima, Flavio V. Loures, José Antonio T. Albuquerque, Marina Barguil Macêdo, Rafael Ribeiro Almeida, Jonas W. Gaiarsa, Luis A. Luévano-Martínez, Thiago Belchior, Meire Ioshie Hiyane, Gordon D. Brown, Marcelo A. Mori, Christian Hoffmann, Marília Seelaender, Willian T. Festuccia, Pedro Manoel Moraes-Vieira, Niels Olsen Saraiva Câmara

**Affiliations:** 1 Department of Immunology, Institute of Biomedical Sciences, University of São Paulo, São Paulo, SP 05508-900, Brazil; 2 Instituto Sírio-Libanês de Ensino e Pesquisa, Hospital Sírio-Libanês, São Paulo, SP 01308-060, Brazil; 3 Division of Endocrinology, Diabetes, and Metabolism, Department of Medicine, Beth Israel Deaconess Medical Center and Harvard Medical School, Boston, MA 02215, USA; 4 Department of Cellular Biology, Institute of Biomedical Sciences, University of São Paulo, São Paulo, SP 05508-900, Brazil; 5 Laboratório Especial de Inovação e Desenvolvimento Industrial, Instituto Butantan, São Paulo, SP 05503-900, Brazil; 6 Tau GC Bioinformatics, Rua Apiacas, 886, São Paulo, SP 05017-020, Brazil; 7 Departamento de Bioquímica, Instituto de Química, Universidade de São Paulo, São Paulo, SP 13565-905, Brazil; 8 Department of Physiology and Biophysics, Institute of Biomedical Sciences, University of São Paulo, São Paulo, SP 05508-900, Brazil; 9 MRC Centre for Medical Mycology, Aberdeen Fungal Group, School of Medicine, Medical Sciences & Nutrition, Institute of Medical Sciences, University of Aberdeen, Aberdeen AB24 3FX, UK; 10 Department of Biochemistry and Tissue Biology, Institute of Biology, University of Campinas, Campinas, SP 13083-970, Brazil; 11 Food Research Center - FoRC, Department of Food Sciences and Experimental Nutrition, School of Pharmaceutical Sciences, University of São Paulo, São Paulo, SP 05508-080, Brazil; 12 Department of Genetics, Evolution and Bioagents, Institute of Biology, University of Campinas, Campinas, SP 13083-970, Brazil; 13 Nephrology Division, Laboratory of Clinical and Experimental Immunology, Federal University of São Paulo, São Paulo, SP 04023-900, Brazil; 14 Department of Medicine, Laboratory of Renal Physiology (LIM 16), University of São Paulo, São Paulo, SP 05403-000, Brazil; 15 Lead Contact

## Abstract

The underlying mechanism by which MyD88 regulates the development of obesity, metainflammation, and insulin resistance (IR) remains unknown. Global deletion of MyD88 in high-fat diet (HFD)-fed mice resulted in increased weight gain, impaired glucose homeostasis, elevated Dectin-1 expression in adipose tissue (AT), and proinflammatory CD11c+ AT macrophages (ATMs). Dectin-1 KO mice were protected from diet-induced obesity (DIO) and IR and had reduced CD11c+ AT macrophages. Dectin-1 antagonist improved glucose homeostasis and decreased CD11c+ AT macrophages in chow- and HFD-fed MyD88 KO mice. Dectin-1 agonist worsened glucose homeostasis in MyD88 KO mice. Dectin-1 expression is increased in AT from obese individuals. Together, our data indicate that Dectin-1 regulates AT inflammation by promoting CD11c+ AT macrophages in the absence of MyD88 and identify a role for Dectin-1 in chronic inflammatory states, such as obesity. This suggests that Dectin-1 may have therapeutic implications as a biomarker for metabolic dysregulation in humans.

## In Brief

Castoldi et al. identify a role for Dectin-1 in obesity and insulin resistance (IR). Dectin-1 KO mice have decreased CD11c+ adipose tissue (AT) macrophages (ATMs) and are protected from obesity and IR. Increased expression of Dectin-1 in AT correlates with obesity.

## Graphical Abstract

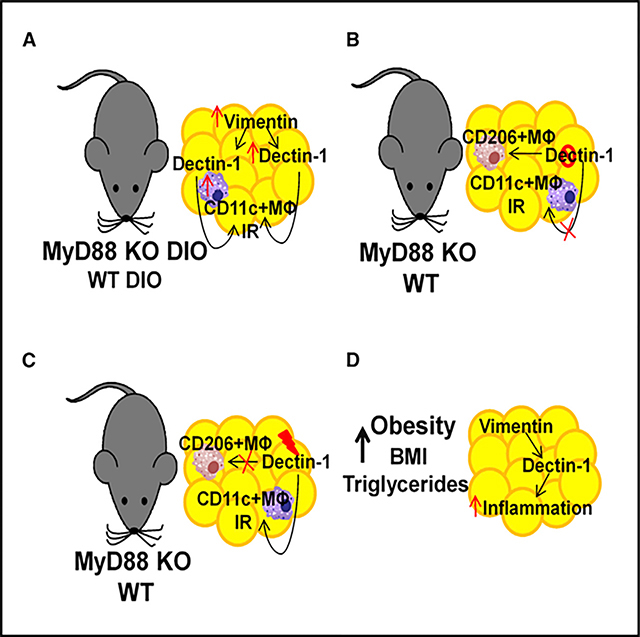

## INTRODUCTION

Obesity is characterized by excessive accumulation of white adipose tissue (AT) due in part to increased food intake, decreased energy expenditure, and changes in lifestyle (Mokdad et al., 2003). According to the World Health Organization (WHO), 39% of adults over 18 years are overweight and 13% are clinically obese (WHO, 2015). A significant number of obese people are also insulin resistant, and obesity is strongly correlated with systemic low-grade inflammation (Belkina and Denis, 2010), highlighting the role of the immune system in the development of insulin resistance (IR).

Innate immune receptors, such as Toll-like receptors (TLRs), are expressed in adipocytes and AT macrophages (ATMs) and are important molecules that orchestrate inflammation-induced IR. TLRs can signal through myeloid differentiation primary response gene 88 (MyD88) (Adachi et al., 1998), an adaptor molecule expressed in immune cells, epithelial cells, and adipocytes (Bhinder et al., 2014; Everard et al., 2014; Hoshi et al., 2012; Yu et al., 2014). Some studies showed that whole-body MyD88-deficient mice develop exacerbated IR in the absence of liver inflammation (Hosoi et al., 2010; Yokoyama et al., 2012). By contrast, retinol binding protein 4 (RBP4) induces IR via a MyD88-dependent pathway (Moraes-Vieira et al., 2016). However, how MyD88 contributes to obesity-induced IR and its actions in specific tissues are still not completely understood.

Dectin-1 is a member of the C-type lectin receptor (CLR) family highly expressed in macrophages and dendritic cells (DCs) (Herre et al., 2004) that recognizes β-glucans at fungal cell walls. Dectin-1 requires interferon regulatory factor 5 (IRF5) for immune responses (del Fresno et al., 2013), and IRF5 is necessary for differentiation of M1 AT macrophages, which play a major role in obesity-induced IR (Krausgruber et al., 2011). Furthermore, Dectin-1 was reported to play a role in tumor cell recognition (Brown, 2006; Chiba et al., 2014). Dectin-1 is activated in macrophages in atherosclerotic plaques by vimentin (Thiagarajan et al., 2013), an intermediate filament expressed in mesenchymal cells (Satelli and Li, 2011).

Altogether, these data suggest that Dectin-1 may be a key factor in the development of obesity-associated inflammation and IR. Because Dectin-1 is expressed in macrophages and DCs, we hypothesized that Dectin-1 modulates AT macrophage function and phenotype, contributing to the development of obesity and IR.

Here, we observed that full deletion of MyD88 is sufficient to cause obesity and IR in mice. However, these phenotypes were partially reversed by depleting MyD88 specifically in the myeloid cell compartment. In the absence of MyD88, we observed that CD11c+ AT macrophages were highly present in the AT, where Dectin-1 was upregulated. Yet, blocking Dectin-1 signaling, in both the lean and diet-induced obese (DIO) mice improved glucose homeostasis and insulin sensitivity. Moreover, Dectin-1-deficient macrophages displayed an anti-inflammatory phenotype, and their conditioned media improved insulin sensitivity in adipocytes. Finally, we found that increased expression of Dectin-1 in the AT from the obese individuals was associated with the enhanced expression of proinflammatory molecules. Thus, our data indicate that Dectin-1 regulates AT inflammation by promoting proinflammatory macrophage polarization in the absence of MyD88. Dectin-1 may be an important therapeutic target for the treatment of the chronic non-resolving inflammation associated with the obese and insulin resistant patients.

## RESULTS

### Absence of MyD88 Signaling Exacerbates Obesity-Induced IR and Polarization of AT Macrophages toward a Proinflammatory Phenotype

**Figure 1.**
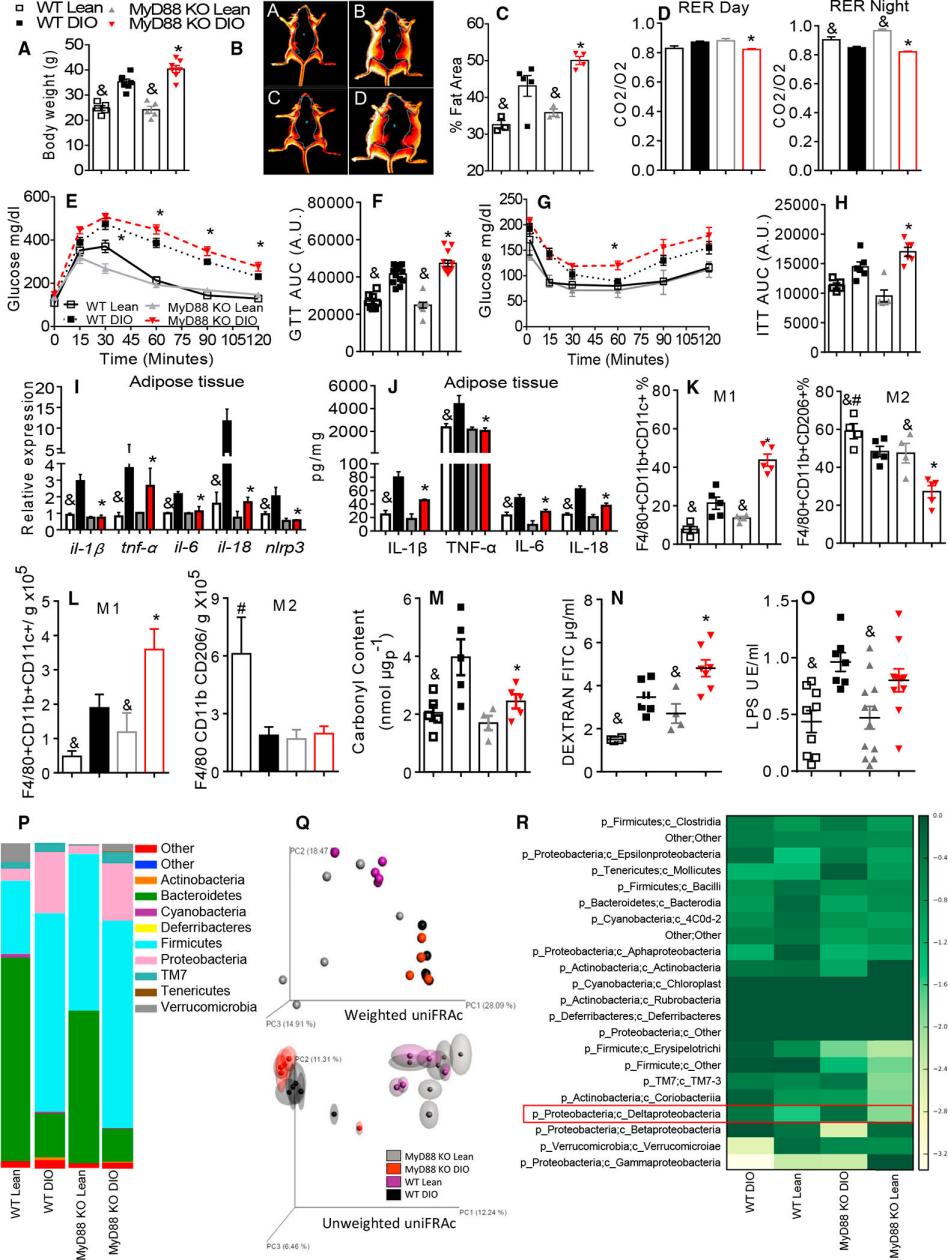
MyD88 Is Necessary for Metabolic Homeostasis (A) Body weight gain at end of diet. (B) Analysis of body fat by DEXA (A, WT lean; B, WT DIO; C, MyD88 KO lean; D, MyD88 KO DIO). (C) Quantification of fat area (% of adiposity). (D) Respiratory exchange ratio (RER) during day and night cycles. (E) Glucose tolerance test (GTT). (F) GTT area under the curve (AUC). (G) Insulin tolerance test (ITT). (H) ITT AUC. (I) AT gene expression of *Il-1β, Tnf-α, Il-6, Il-8*, and *Nlrp3*. (J) AT protein quantification of IL-1β, TNF-α, IL-6, and IL-18. (K) Frequency of F4/80+CD11b+CD11c+ and F4/80+CD11b+CD206+ AT macrophages. (L) Absolute numbers of F4/80+CD11b+CD11c+ and F4/80+CD11b+CD206+ AT macrophages/g of AT. (M) AT quantification of carbonyl content. (N) Serum quantification of dextran fluorescein isothiocyanate (FITC). (O) Serum quantification of LPS. (P) Relative abundance of major commensal bacteria phyla in feces. (Q) Principal coordinate (PC) analysis of gut microbiota composition based on weighted (upper) and unweighted (lower) UniFRAc in WT and MyD88 KO, lean, and DIO mice. (R) Heatmap based on relative abundance of OTUs in lean and DIO WT and MyD88 KO mice at genus level. *p ≤ 0.05 versus WT DIO; &p ≤ 0.05 versus DIO on same genotype; #p ≤ 0.05 versus all groups n = 5–8 mice. The data are represented as mean ± SEM.

MyD88 signaling is a critical component for TLR signaling. Although MyD88 has been studied in the context of obesity (Hosoi et al., 2010; Yokoyama et al., 2012), there are still some unsolved questions to be accessed. DIO MyD88 knockout (KO) mice gained more weight and had increased adiposity when compared to the DIO wild-type (WT) animals (Figures 1A–1C and S1A). DIO MyD88 KO mice had decreased VO2 and VCO2 and were more glucose intolerant and insulin resistant (Figures 1D–1H, S1B, and S1C). Moreover, insulin-stimulated AT Ser473AKT (serine 473 protein-kinase B) phosphorylation (Figure S1D), glucose transporter type 4 (GLUT-4) protein expression (Figure S1D), ex vivo AT glucose uptake, and glucose oxidation (Figure S1E) were all decreased in the DIO MyD88 KO mice. Together, these data demonstrate that MyD88 is necessary for the maintenance of systemic glucose homeostasis.

Next, we investigated whether the absence of MyD88 would affect the obesity-induced AT inflammation. DIO MyD88 KO mice had decreased AT gene expression of *Il-1β, Tnf-α, Il-6, Il-18*, and *Nlrp3* (Figure 1I). Protein levels of interleukin 1 beta (IL-1β), tumor necrosis factor alpha (TNF-α), interleukin 6 (IL-6), and interleukin 18 (IL-18) were also decreased in the AT (Figure 1J) and serum (Figure S1F). Phosphorylation of c-Jun N-terminal kinase (JNK) and IκB-α (nuclear factor of kappa light polypeptide gene enhancer in B-cells inhibitor, alpha) in the AT of the DIO MyD88 KO mice were lower compared to the DIO WT mice (Figure S1G). We next characterized the phenotype of the epididymal AT macrophages. We defined proinflammatory macrophages as the CD11c+ AT macrophages and anti-inflammatory macrophages as the mannose receptor (cluster of differentiation 206) (CD206+) AT macrophages, as previously described (Moraes-Vieira et al., 2014). DIO MyD88 KO mice had increased total numbers as well as percentage of the CD11c+ AT macrophages and decreased numbers and percentages of the anti-inflammatory CD206+ AT macrophages when compared to the DIO WT mice (Figures 1K, 1L, and S1H). Similar results were observed in liver (Figures S1I–S1Q). AT carbonyl content was decreased in the DIO MyD88 KO mice (Figure 1M). This suggests that the DIO MyD88 KO mice have reduced levels of oxidative injury. Together, these data indicate that loss of MyD88 in the DIO mice decreases AT inflammation despite increased levels of the CD11c+ AT macrophages.

In obesity, alterations in gut microbiota composition are associated with increased systemic lipopolysaccharide (LPS) levels, which contribute to AT inflammation and the development of IR (Castoldi et al., 2015a; Ley et al., 2005). DIO MyD88 KO mice had increased gut permeability (Figures 1N and S2A–S2H) and circulating LPS levels compared to the lean MyD88 KO mice (Figure 1O), but were not different when compared to the DIO WT mice. Concomitantly, gut inflammatory markers, defensins, and anti-microbial peptides were decreased in the DIO MyD88 KO mice (Figures S2I–S2Q). High-fat diet (HFD)-fed mice have an increased abundance of *Firmicutes* and reduced *Bacteroidetes* in the feces (Cani et al., 2008). As reported, the abundance of *Bacteroidetes* was decreased in both the DIO WT and MyD88 KO mice, whereas *Firmicutes* was increased compared to the lean animals (Figure 1P). Next, comparisons between the groups were made using principal coordinate analysis (PCA) based on weighted and unweighted UniFRAc distances. A change in the microbiota was detected when the WT and MyD88 KO mice were fed an HFD, which can be visualized by the tight clustering of the microbial communities on axis principal coordinate 1 (PC1) (Figures 1Q, S1R, and S1S). No differences were found between the mouse genotypes (Table S2). We found increased *Deltaproteobacteria* abundance in both DIO mice (Figure 1R) and *Desulfovibrio* was also increased in both DIO mice (Figure S7A), and its abundance is related to type 2 diabetes (Qin et al., 2012). Thus, HFD modulates gut microbiota diversity in both the WT and MyD88 KO mice when compared to the controls. However, there was no difference in microbiota diversity between the MyD88 KO and WT mice after 90 days on an HFD.

### MyD88 Expression in Myeloid Cells, but Not in Adipocytes, Is Necessary for the Development of Obesity-Induced IR, AT Inflammation, and Proinflammatory AT Macrophage Polarization

Although several cell types can signal through MyD88, their individual contribution to obesity-associated inflammation and metabolic deregulation is still unclear. Therefore, we investigated the cell types in which MyD88 is necessary for the development of obesity-induced inflammation and IR.

**Figure 2.**
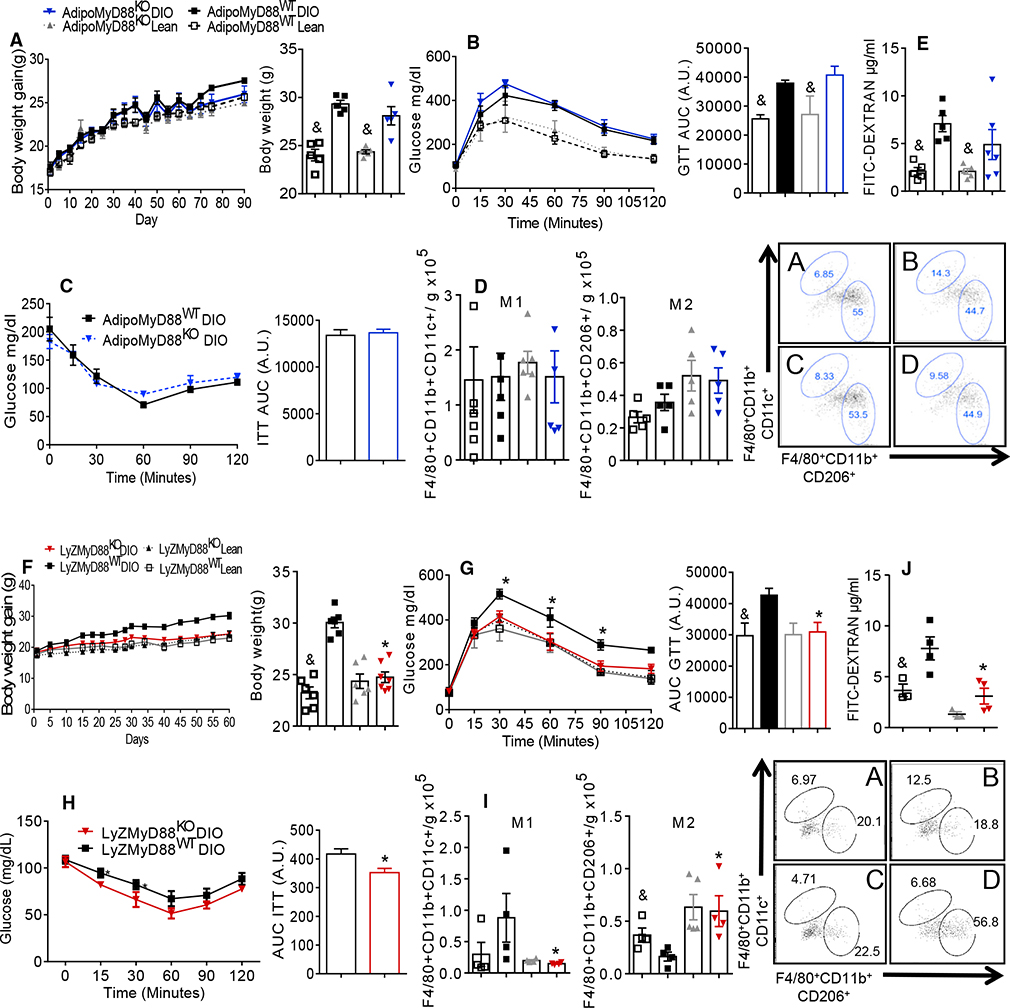
MyD88 Depletion in Myeloid Cells, but Not in AT, Protects Mice from Obesity Development and IR (A) AdipoMyD88^KO^ studies: body weight gain and body weight at the end of diet. (B) Glucose tolerance test (GTT) and area under the curve (AUC). (C) Insulin tolerance test (ITT) and AUC. (D) Absolute numbers of F4/80+CD11b+CD11c+ and F4/80+CD11b+CD206+ AT macrophages/g of AT (A, AdipoMyD88^WT (Control)^ lean; B, AdipoMyD88^WT (Control)^ DIO; C, AdipoMyD88^KO^ lean; D, AdipoMyD88^KO^ DIO). (E) Serum FITC dextran levels. (F) LyZMyD88KO studies: body weight gain and body weight at the end of diet. (G) GTT and the respective AUC. (H) ITT and the respective AUC. (I) Absolute numbers of F4/80+CD11b+CD11c+ and F4/80+CD11b+CD206+ AT macrophages/g of AT (A, LyZMyD88^WT (Control)^ lean; B, LyZMyD88^WT (Control)^ DIO; C, LyZMyD88^KO^ lean; D, LyZMyD88^KO^ DIO). (J) Serum FITC dextran levels. *p ≤ 0.05 versus MyD88^WT^ DIO; &p ≤ 0.05 versus DIO on same genotype; #p ≤ 0.05 versus all groups. The data are represented as mean ± SEM.

HFD-fed AdipoMyD88^KO^ mice had similar body weights, AT weight, glucose tolerance, and IR compared to the control DIO AdipoMyD88^WT^ mice (Figures 2A–2C and S3B). There was no difference in the respiratory exchange ratio (RER) between the adipose-specific MyD88 KO mice and their controls (Figure S3C). AT inflammation was also not different between the DIO AdipoMyD88^KO^ and DIO AdipoMyD88^WT^ mice (Figures 2D and S3D–S3G). Intestinal permeability was increased in both the DIO AdipoMyD88^KO^ and DIO AdipoMyD88^WT^ mice compared to their lean controls (Figure 2E). Gut permeability was not different between the HFD-fed AdipoMyD88 KO and the WT controls (Figure 2E). These data indicate that MyD88 expression in adipocytes does not contribute to the obesity-induced AT inflammation, IR, or intestinal permeability.

We next hypothesized that MyD88 expression in macrophages may drive the proinflammatory and metabolic phenotypes seen in the global MyD88 deletion in mice. Similar to what was previously reported by Yu et al. (2014), the DIO LyZMyD88^KO^ mice were leaner (Figures 2F and S3K), had improved glucose tolerance, and insulin sensitivity (Figures 2G and 2H) without changes in RER when compared to the DIO LyZMyD88^WT^ mice (Figure S3L). Also, the DIO LyZMyD88^KO^ mice had reduced AT inflammation (Figures S3M and S3N), with decreased numbers and percentages of the proinflammatory CD11c+ AT macrophages compared to the controls on the same diet (Figures 2I and S3O). The numbers and percentages of the anti-inflammatory CD206+ AT macrophages were markedly increased in the DIO LyZMyD88^KO^ mice (Figures 2I and S3P) compared to the DIO controls. DIO LyZMyD88^KO^ mice had reduced gut permeability compared to the DIO LyZMyD88^WT^ (Figure 2J). These results indicate that myeloid expression of MyD88 is required for obesity-induced inflammation, control of energy expenditure, and subsequent IR development. However, our finding that the global MyD88 KO mice were heavier and IR when compared to the tissue-specific MyD88 KO animals remained unclear. Thus, we hypothesized that another molecule may be involved in this intricate network to drive metabolic dysregulation.

### Dectin-1 Levels Are Increased in AT and in AT Macrophages of MyD88 KO Mice

**Figure 3.**
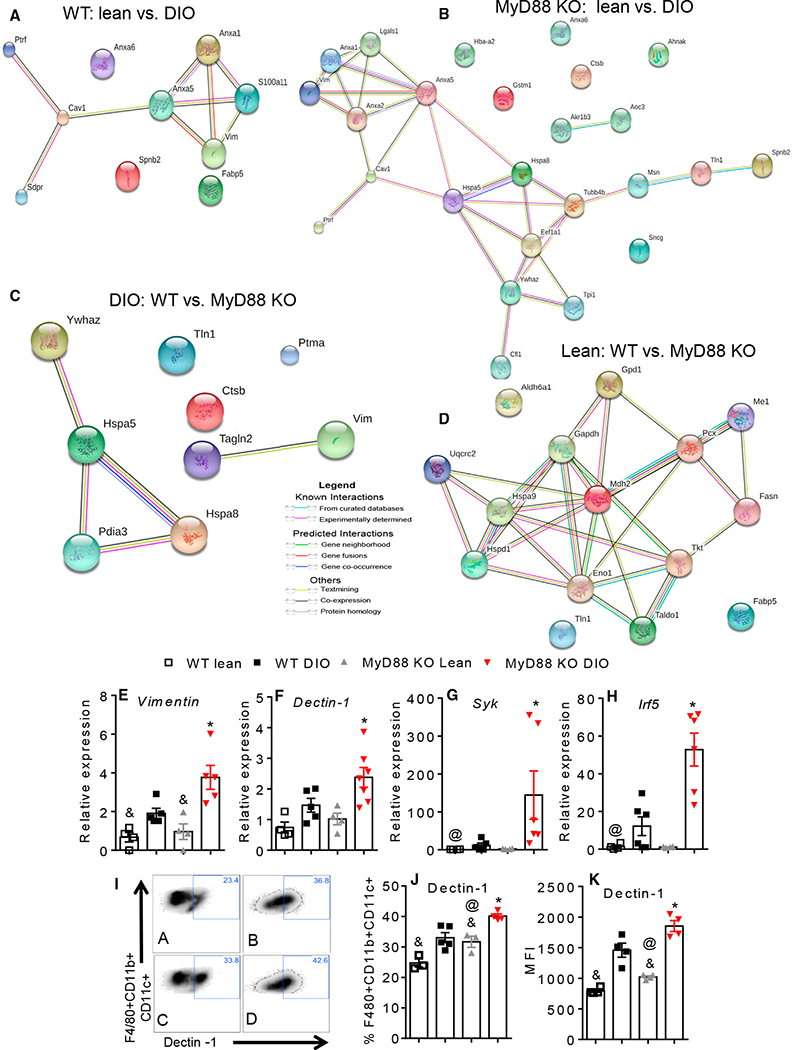
Absence of MyD88 Augments Dectin-1 Expression in AT Macrophages (A) Proteomics analysis in AT showing upregulated proteins in DIO WT versus lean WT (STRING network view: colored lines between the proteins indicate the various types of interaction evidence). (B) DIO MyD88 KO versus lean MyD88 KO. (C and D) DIO MyD88 KO versus DIO WT (C) and lean MyD88 KO versus lean WT (D). (E) AT gene expression of (Figure 1R), *Vimentin*. (F–H) AT gene expression of *Dectin-1* (F), *Syk* (G), and *Irf-5* (H). (I) Representative flow cytometry analysis of Dectin-1 in AT macrophages F4/80+CD11b+CD11c+ from WT and MyD88 KO, lean, and DIO mice (A: WT lean; B: WT DIO; C: MyD88 KO lean; D: MyD88 KO DIO). (J) Dectin-1 expression in AT macrophages F4/80+CD11b+CD11c+ from WT and MyD88 KO, lean, and DIO mice. (K) Median fluorescence intensity (MFI) of Dectin-1 expression in F4/80+CD11b+CD11c+ AT macrophages. *p ≤ 0.05 versus WT DIO; &p ≤ 0.05 versus DIO on same genotype; @p ≤ 0.05 versus WT lean. The data are represented as mean ± SEM.

First, we performed a proteomic analysis to identify differentially expressed proteins in the AT of obese animals. We found that molecules related to lipid metabolism were differentially expressed. Vimentin was significantly upregulated in the AT of the DIO WT mice (Figures 3A and S4A), as well as in the DIO global MyD88 KO mice (Figures 3B and S4B) compared to the lean mice. Moreover, vimentin was also upregulated in the DIO MyD88 KO mice compared to the DIO WT mice (Figures 3C and S4C), but no difference was observed between the WT and MyD88 KO lean mice (Figures 2D and S4C). Gene expression of *Vimentin* was consistently increased in the MyD88 KO mice compared to the WT mice on the HFD (Figure 3E).

Since vimentin was previously associated to Dectin-1 (Thiagarajan et al., 2013), and may act as an endogenous ligand for Dectin-1, worsening IR in our model, we next analyzed the expression of Dectin-1 in the AT of the WT and MyD88 KO mice. *Dectin-1* mRNA expression was increased in the AT of the DIO MyD88 KO mice (Figure 3F). Moreover, the Dectin-1 adaptor molecule *Syk* (spleen tyrosine kinase) and the transcription factor *Irf5* were also increased in the DIO MyD88 KO mice (Figures 3G and 3H). *Dectin-1* and *Syk* were similarly increased in the DIO AdipoMyD88^KO^ and the DIO AdipoMyD88^WT^ mice (Figures S3H and S3I), while decreased in the DIO LyZMyD88^KO^ compared to the DIO LyZMyD88^WT^ (Figures S3Q and S3R). Dectin-1 expression was upregulated in the CD11c+ AT macrophages from the epididymal AT of the DIO MyD88 KO compared to the DIO WT mice, and it was also higher in the CD11c+ AT macrophage of the lean MyD88 KO mice (Figures 3I–3K). These data indicate that Dectin-1 expression is elevated during obesity and in the global deficiency of MyD88 in mice. Therefore, Dectin-1 may contribute to the development of IR and induction of proinflammatory AT macrophage polarization.

### Blockade of Dectin-1 Suppresses IR and CD11c+ AT Macrophage Polarization

**Figure 4.**
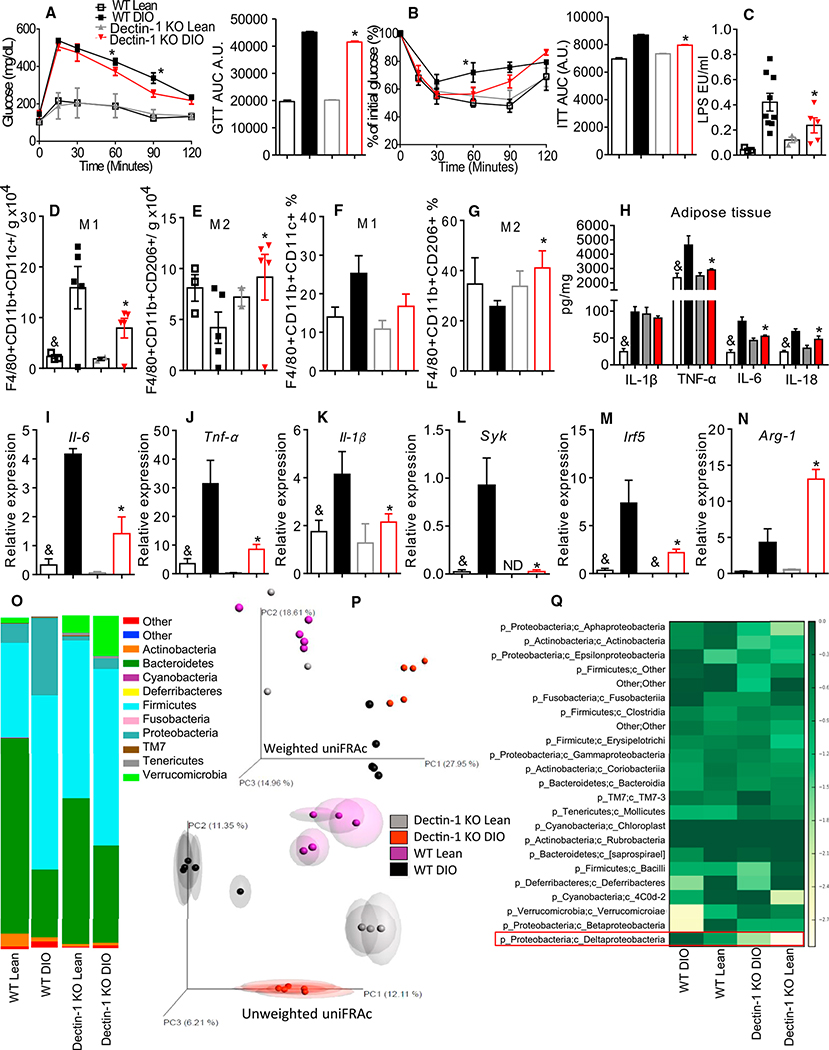
Dectin-1 KO Mice Are Protected from Metabolic Syndrome (A) Glucose tolerance test (GTT) and area under the curve (AUC). (B) Insulin tolerance test (ITT) and AUC. (C) Serum LPS quantification. (D) Absolute number of F4/80+CD11b+CD11c+ AT macrophages/g of AT. (E) Absolute numbers of F4/80+CD11b+CD206+ AT macrophages/g of AT. (F) Frequency of F4/80+CD11b+CD11c+ AT macrophages. (G) Frequency of F4/80+CD11b+CD206+ AT macrophages. (H) AT protein quantification of IL-1β, TNF-α, IL-6, and IL-18. (I–N) AT gene expression of *Il-1β* (I), *Tnf-α* (J), *Il-6* (K), *Syk* (L), *Irf5* (M), and *Arginase-1* (N), respectively. (O) Relative abundance of major commensal bacteria phyla in feces. (P) Principal coordinate (PC) analysis of gut microbiota composition based on weighted (upper) and unweighted (lower) UniFRAc in WT and Dectin-1 KO, lean, and DIO mice. (Q) Heatmap based on relative abundance of OTUs in lean and DIO WT and Dectin-1 KO mice at genus level. *p ≤ 0.05 versus WT DIO; &p ≤ 0.05 versus DIO on same genotype; #p ≤ 0.05 versus all groups n = 5–8 mice. The data are represented as mean ± SEM.

Because Dectin-1 expression is increased in the DIO mice, we asked whether inhibition of Dectin-1 expression and/or signaling could improve the AT inflammation, glucose intolerance, and IR. HFD-fed Dectin-1 KO mice had improved glucose tolerance and insulin sensitivity (Figures 4A and 4B). Chow- and HFD-fed Dectin-1 KO mice displayed decreased numbers of the proinflammatory CD11c+ AT macrophages (Figures 4D–4F) and increased numbers of the anti-inflammatory CD206+ AT macrophages (Figures 4F and 4G). DIO Dectin-1 KO mice had decreased gene and protein expression of the proinflammatory cytokines in the AT (Figures 4H–4K). *Syk* and *Irf-5* mRNA expression were decreased and *Arginase-1* was increased in the DIO Dectin-1 KO mice (Figures 4L–4N). Gut microbiota analysis showed decreased *Proteobacteria* and increased *Bacteroidetes* in the DIO Dectin-1 KO mice compared to the DIO WT mice (Figure 4O). Moreover, bacterial proportions and communities in the lean and DIO Dectin-1 KO mice were distinctly separated from the lean and DIO WT mice on the PC1 and PC2 axes (Figures 4P, S1S, and S1T; Table S3). The abundance of *Deltaproteobacteria* was decreased in the DIO Dectin-1 KO mice compared to the DIO WT mice and absent in the lean Dectin-1 KO mice (Figure 4Q). *Desulfovibrio* was not detected in the Dectin-1 KO mice (Figure S7B). These data support our hypothesis that Dectin-1 contributes to the development of obesity and IR by regulating the CD11c+ AT macrophage polarization and by modulating gut microbiota populations of the HFD-fed mice.

**Figure 5.**
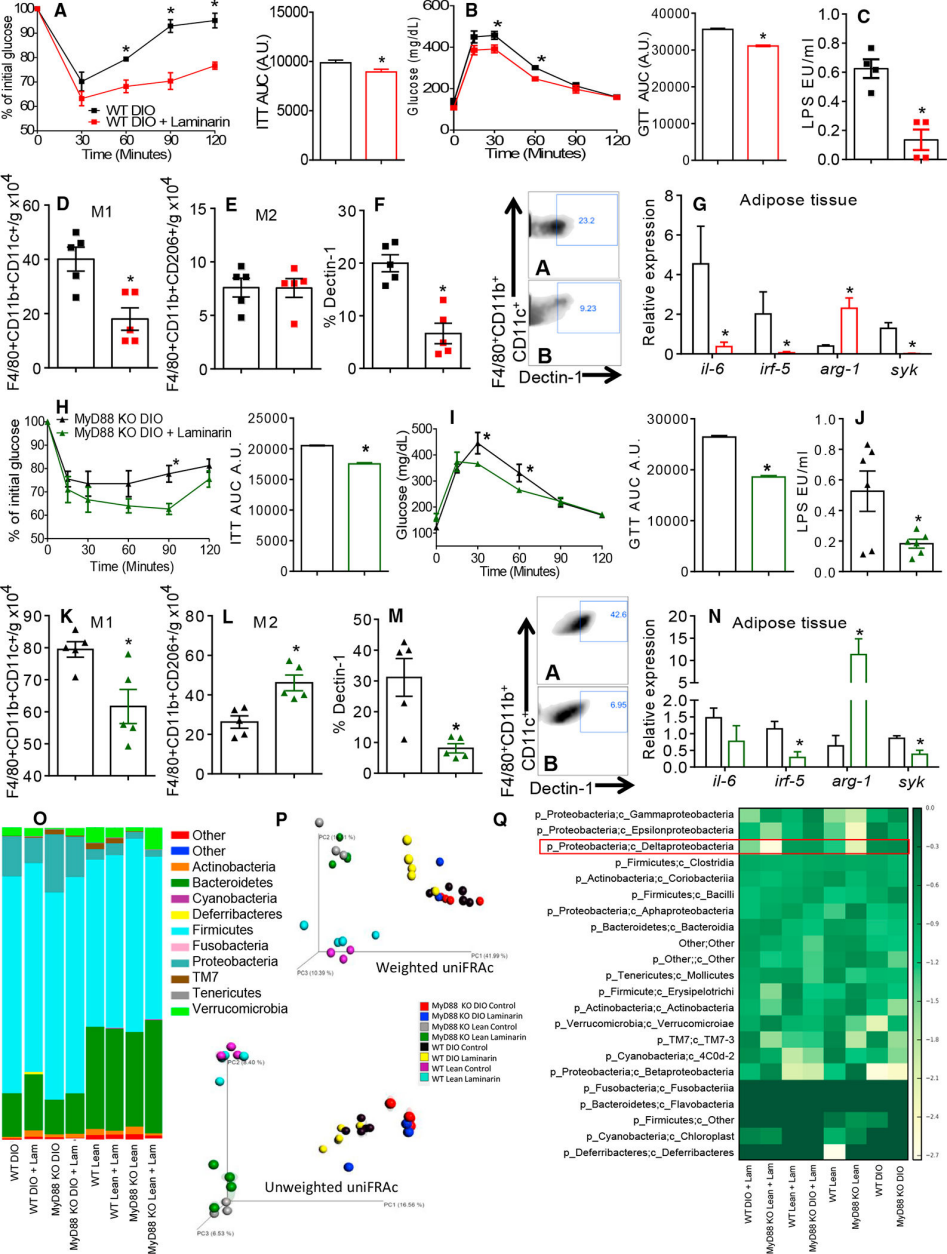
Dectin-1 Antagonist Treatment Protects Mice from Developing IR and Reduces CD11c+ AT Macrophages Polarization (A) Insulin tolerance test (ITT) and area under the curve (AUC) of DIO WT mice control (vehicle-treated) and 5 weeks treated with laminarin. (B) Glucose tolerance test (GTT) and AUC. (C) Serum LPS quantification. (D) Total numbers of F4/80+CD11b+CD206+ AT macrophages/g of AT. (E) Total numbers of F4/80+CD11b+CD206+ AT macrophages/g of AT. (F) Expression of Dectin-1 in F4/80+CD11b+CD11c+ AT macrophages (A, WT DIO + PBS; B, WT DIO + Laminarin). (G) AT gene expression of *Il-6, Irf-5, Arginase-1*, and *Syk*. (H) ITT and AUC of DIO MyD88 KO mice control (vehicle-treated) and 5 weeks treated with laminarin. (I) GTT and AUC. (J) Serum LPS quantification. (K) Total numbers of F4/80+CD11b+CD11c+ AT macrophages/g of AT. (L) Total numbers of F480+CD11b+CD206+ AT macrophages/g of AT. (M) Expression of Dectin-1 in F4/80+CD11b+CD11c+ AT macrophages (A, MyD88 KO DIO + PBS; B, MyD88 KO DIO + Laminarin). (N) AT gene expression of *Il-6, Irf-5, Arginase-1*, and *Syk*. (O) Relative abundance of major commensal bacteria phyla in feces. (P) Principal coordinate (PC) analysis of gut microbiota composition based on weighted (upper) and unweighted (lower) UniFrac in lean and DIO WT and MyD88 KO laminarin- and vehicle-treated mice. (Q) Heatmap based on relative abundance of OTUs in lean and DIO WT and MyD88 KO, laminarin- and vehicle-treated mice at genus level. *p < 0.05 versus C57BL/6 + PBS, n = 5 each group. The data are represented as mean ± SEM.

Next, we treated the chow- and HFD-fed WT and MyD88 KO mice with laminarin or vehicle (PBS). Laminarin is a soluble β-glucan from seaweed and binds to Dectin-1 without stimulating downstream signaling pathways (Brown et al., 2002; Gersuk et al., 2006; Huang et al., 2012). After 5 weeks of treatment, both the DIO and lean mice displayed improved insulin sensitivity and glucose tolerance (Figures 5A, 5B, 5H, 5I, S5A, S5B, S5H, and S5I). Laminarin reduced the total numbers of the CD11c+ AT macrophages in the MyD88 KO and WT mice (Figures 5D, 5K, S5D, and S5K) without affecting the numbers of the CD206+ AT macrophages in the DIO or lean WT mice (Figures 5E and S5E). However, the numbers of the CD206+ AT macrophages in both the lean and DIO MyD88 KO mice were increased (Figures 5L and S5L). Laminarin reduced Dectin-1 expression in the CD11c+ AT macrophages from the WT and MyD88 KO on the chow and HFD fed mice (Figures 5F, 5M, S5F, and S5M). Furthermore, gene expression of *Il-6* was decreased in both the chow- and HFD-fed WT mice, but no difference was observed in both the laminarin-treated DIO and lean MyD88 KO mice (Figures 5G, 5N, S5G, and S5N). We found reduced AT *Irf5* and *Syk* gene expression in all the laminarin-treated mice compared to vehicle-treated mice (Figures 5G, 5N, S5G, and S5N). In both lean and DIO WT and MyD88 KO mice, AT expression of *Arginase-1* was increased after the laminarin treatment compared to the vehicle mice (Figures 5G, 5N, S5G, and S5N). Thus, inhibition of Dectin-1 protects mice from IR, and this is associated with decreased proinflammatory CD11c+ AT macrophages.

We next determined the effect of laminarin on the gut microbiota abundance and diversity in the lean and DIO WT and MyD88 KO mice. We did not observe an effect of laminarin on phyla abundance in the DIO and lean mice compared to the vehicle-control mice (Figure 5O). There were changes in bacterial proportions and communities in the WT and MyD88 KO mice when comparing the diet and genotypes after 5 weeks of HFD feeding (Figure 5P; Table S4). However, laminarin treatment did not induce changes compared to the vehicle-treated mice (Figures 5P, S5O, and S5P; Table S4). Blockade of Dectin-1 with laminarin-induced small changes in Deltaproteobacteria operational taxonomic units (OTUs) (Figure 5Q). Altogether, these data suggest that Laminarin treatment does not induce gut microbiota changes after 5 weeks of the HFD feeding and the laminarin effects are independent of the gut microbiota alterations.

We performed internal transcribed spacer (ITS) sequencing on the feces from both the lean and DIO MyD88 KO and WT mice to rule out any type of fungal response or dependency. We did not detect fungi in the feces of the laminarin- or vehicle-treated mice (Figure S2U). Thus, blocking Dectin-1 results in beneficial effects on the inflammation and glucose homeostasis, after 5 weeks of treatment, without affecting the gut microbiota.

### Dectin-1 Agonist Treatment Worsens IR and CD11c+ AT Macrophage Polarization

**Figure 6.**
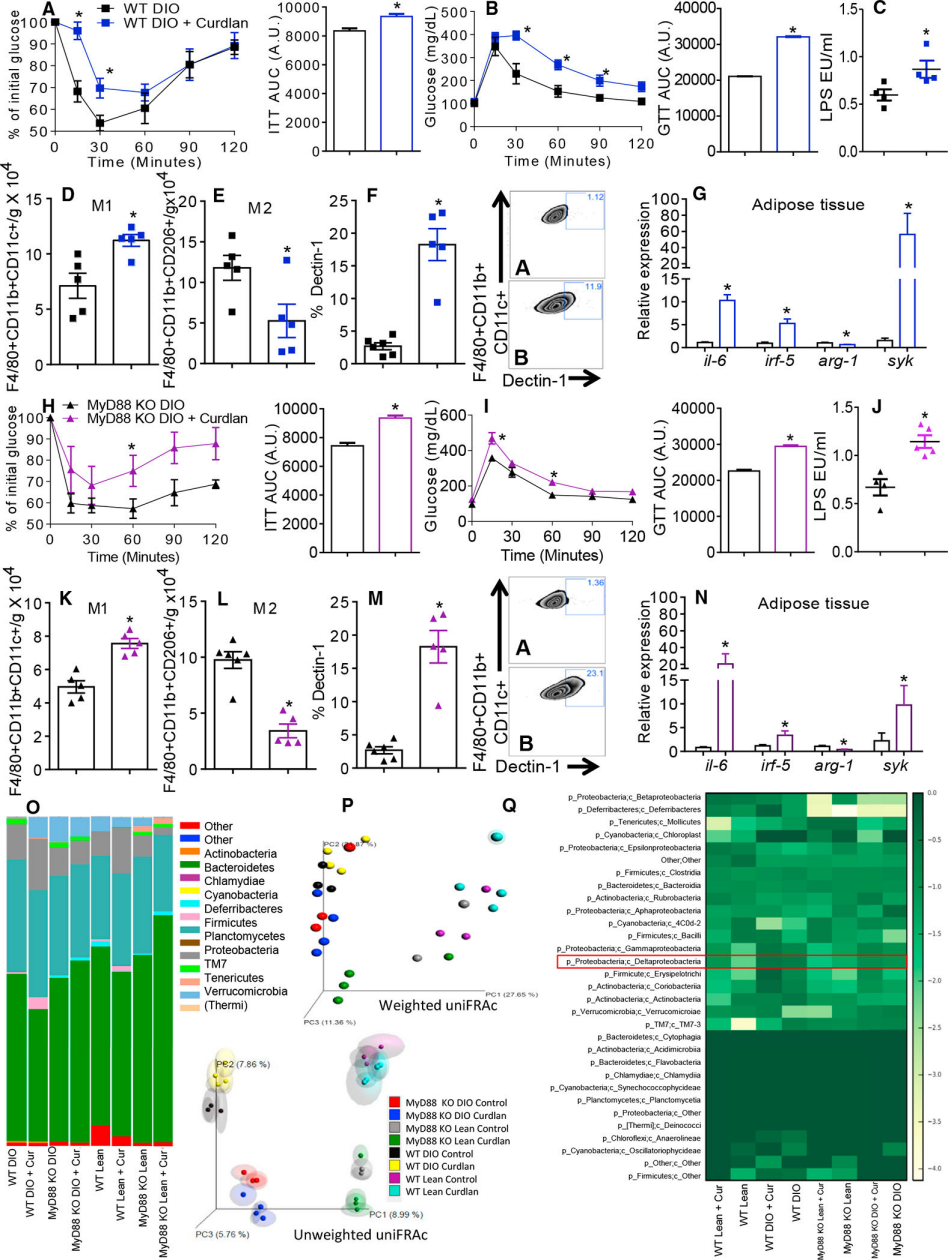
Dectin-1 Agonist Treatment Increases IR and CD11c+ AT Macrophages Polarization (A) Insulin tolerance test (ITT) and area under the curve (AUC) of DIO WT mice control (vehicle-treated) and 5 weeks treated with curdlan. (B) Glucose tolerance test (GTT) and AUC. (C) Serum LPS quantification. (D) Total numbers of F4/80+CD11b+CD11c+ AT macrophages/g of AT. (E) Total numbers of F4/80+CD11b+CD206+ AT macrophages/g of AT. (F) Expression of Dectin-1 in F4/80+CD11b+CD11c+ AT macrophages (A, WT DIO + PBS; B, WT DIO + curdlan). (G) AT gene expression of *Il-6, Irf-5, Arginase-1*, and *Syk*. (H) ITT and AUC of DIO MyD88 KO mice control (vehicle-treated) and 5 weeks treated with curdlan. (I) GTT and AUC. (J) Serum LPS quantification. (K) Total numbers of F4/80+CD11b+CD11c+ AT macrophages/g of AT. (L) Total numbers of F4/80+CD11b+CD206+ AT macrophages/g of AT. (M) Expression of Dectin-1 in F4/80+CD11b+CD11c+ AT macrophages (A, MyD88 KO DIO + PBS; B, MyD88 KO DIO + curdlan). (N) AT gene expression of *Il-6, Irf-5, Arginase-1*, and *Syk*. (O) Relative abundance of major commensal bacteria phyla in feces. (P) Principal coordinate (PC) analysis of gut microbiota composition based on weighted (upper) and unweighted (lower) UniFrac in WT and MyD88 KO curdlan- and vehicle-treated mice. (Q) Heatmap based on relative abundance of OTUs in lean and DIO WT and MyD88 KO, curdlan-, and vehicle-treated mice at genus level. *p < 0.05 versus C57BL/6 + PBS, n = 5 each group. The data are represented as mean ± SEM.

Since Dectin-1 antagonism prevented AT macrophage polarization toward a proinflammatory profile, we asked whether Dectin-1 activation could worsen IR. Curdlan-treated lean and DIO MyD88 KO and WT mice had worsened insulin sensitivity and glucose tolerance (Figures 6A, 6B, 6H, 6I, S6A, S6B, S6H, and S6I) and increased serum LPS levels (Figures 6C, 6J, S6C, and S6J). Curdlan increased the numbers of the CD11c+ AT macrophages (Figures 6D, 6K, S6D, and S6K) and decreased the number of the CD206+ AT macrophage compared to the untreated mice (Figures 6E, 6L, S6E, and S6L). Curdlan treatment in the lean and DIO WT and MyD88 KO mice increased expression of Dectin-1 in the CD11c+ AT macrophages (Figures 6F, 6M, S6F, and S6M) and augmented expression of *Il-6, Irf5, Arg-1*, and *Syk* in the AT (Figures 6G, 6N, S6G, and S6N).

We next asked if curdlan treatment could alter the gut microbiota. Curdlan treatment did not change the abundances of *Bacteroidetes* or *Firmicutes* compared to the vehicle-treated DIO and lean animals (Figure 6O). PC analysis showed differences among the groups in diet and genotype, after 5 weeks of treatment (Figure 6P; Table S5). A trend toward clustering of samples from the curdlan-treated and vehicle-treated groups was observed, especially using weighted UniFRAc distances, however, no statistically significant differences were found (Figures 6P, S6O, and S6P; Table S5). OTU analysis showed that activation of Dectin-1 by curdlan did not restore *Deltaproteobacteria* (Figure 6Q). These data indicate that Dectin-1 activation induced the CD11c+ AT macrophage polarization, increased AT inflammation, and impaired glucose homeostasis without changing the gut microbiota after 5 weeks of treatment.

### Dectin-1 Increases M1 Macrophages Polarization and Increases Insulin Sensitivity in Adipocytes In Vitro

**Figure 7.**
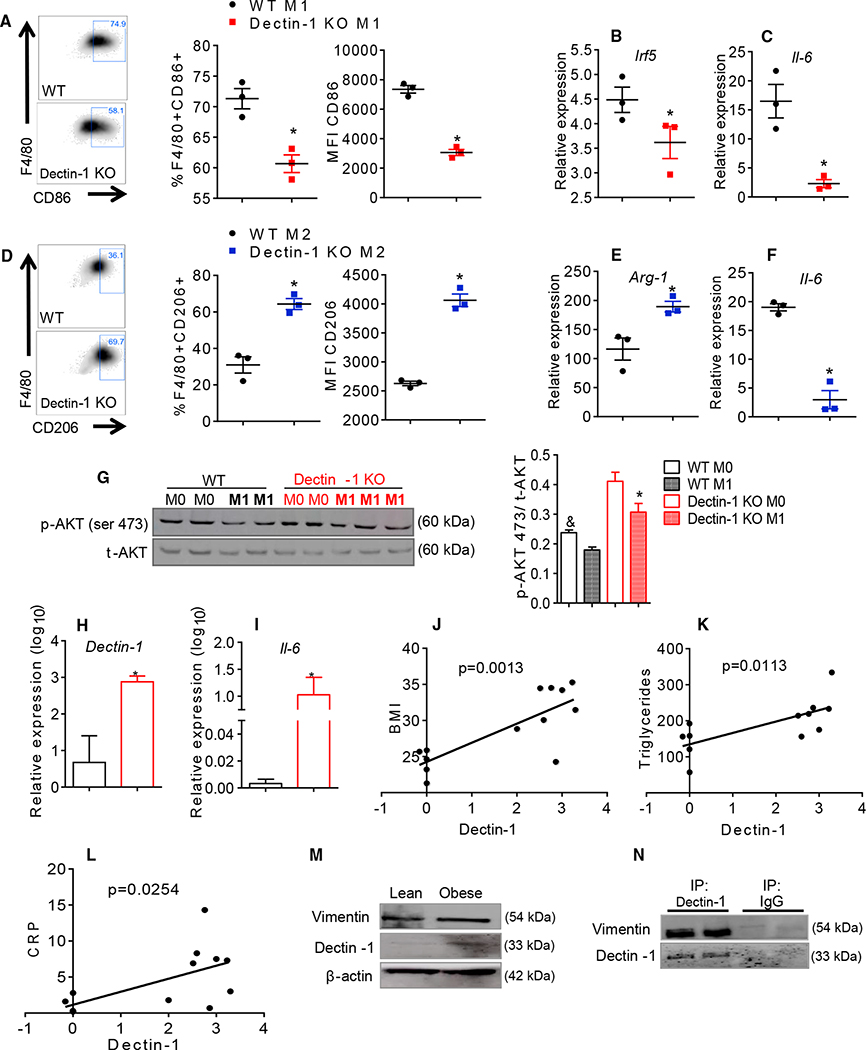
Dectin-1 Is Correlated to Proinflammatory Macrophages Polarization and Obesity in Humans (A) CD86 expression in polarized M1 BMDMs (IFN-γ + LPS) from WT and Dectin-1 KO mice. (B and C) Gene expression of *irf-5* (B) and *il-6* in M1 macrophages (C). (D) CD206 expression in polarized M2 BMDMs (IL-4 + IL-13) from WT and Dectin-1 KO mice. (E and F) Gene expression of *Arginase-1* (E) and *Il-6* in M2 macrophages (F). (G) Adipocytes p-AKT ser473 quantification after 24 hr cultured with M0 and M1 BMDMs media from WT and Dectin-1 KO mice. (H and I) Analysis of genic expression of *Dectin-1* (H) and *Il-6* in mesenteric AT from obese and lean individuals (I). (J) Analysis of the correlation between Dectin-1 gene expression and BMI values. (K) Analysis of the correlation between Dectin-1 gene expression and triglycerides levels. (L) Analysis of the correlation between Dectin-1 gene expression and CRP. (M) Protein expression of vimentin and Dectin-1 in AT from lean and obese humans. (N) Human AT co-immunoprecipitation of Dectin-1 and western blotting showing its interaction with vimentin. For BMDMs studies, *p ≤ 0.05, n = 3 each group. For human studies, *p ≤ 0.05 versus lean individuals, n = 6–7 each group. The data are represented as mean ± SEM.

To confirm the role of Dectin-1 on macrophage polarization, we differentiated bone marrow-derived macrophages (BMDMs) from the Dectin-1 KO and WT mice. Dectin-1 KO BMDMs had decreased M1 polarization under LPS and interferon (IFN)-γ stimulation (Figure 7A) and expressed lower levels of *Irf5* and *Il-6* mRNA (Figures 7B and 7C). Dectin-1 KO BMDMs stimulated with the IL-4 and IL-13 polarized toward an M2 phenotype and had reduced *Il-6* and increased *Arginase*-1 gene expression (Figures 7D and 7F). We also tested the capacity of the M1 BMDMs from the Dectin-1 KO mice to impair insulin signaling in adipocytes. 3T3-L1 adipocytes differentiated in vitro, were cultured during 24 hr with conditioned media from activated M1 BMDMs from the WT and Dectin-1 KO mice. Ser473AKT phosphorylation was increased in adipocytes treated with the Dectin-1 KO-conditioned media compared to the adipocytes treated with conditioned media from the WT M1 BMDMs (Figure 7G). These data suggest that Dectin-1 expression drives M1 macrophages polarization and may contribute to impaired insulin sensitivity in adipocytes in vitro.

### AT from Obese Individuals Displays Increased Dectin-1 Expression

We next aimed to determine whether the expression level of Dectin-1 in the AT from obese humans (Table S1) correlates with the parameters of systemic metabolic dysregulation and inflammation. Dectin-1 gene and protein expression were increased in the mesenteric AT of obese individuals compared to the lean controls (Figures 7H and 7M). Similarly, *Il-6* expression was increased in the obese AT (Figure 7I). Dectin-1 expression positively correlated with BMI, serum triglycerides, and C reactive protein (CRP) levels in the obese individuals (Figures 7J–7L). In addition, vimentin was increased in the AT from the obese individuals (Figure 7M). Co-immunoprecipitation of Dectin-1 and vimentin from the AT of obese individuals showed a direct interaction between these two proteins (Figure 7N). These data indicate that Dectin-1 expression is increased in obesity and influences the AT IR in humans.

## DISCUSSION

The expression of the proinflammatory cytokines in the AT is increased in models of HFD-induced obesity (Castoldi et al., 2015b; Hotamisligil et al., 1996; Trayhurn, 2005). Proinflammatory CD11c+ macrophages also accumulate in the AT of obese mice (Amano et al., 2014; Oh et al., 2012; Shaul et al., 2010). Innate immune receptors can sense several alarmins in the obese state, which contributes to the initiation of the inflammatory response (Moraes-Vieira et al., 2016). The MyD88 pathway is a central component of this immune response.

We show that the whole-body MyD88 KO mice fed an HFD are heavier and more insulin resistant without increased systemic or AT inflammation. This suggests that the global MyD88 signaling contributes to regulating the immune response in obesity, but may have differential roles in a tissue-dependent manner. MyD88 signaling plays an important role in fibrosis and atherosclerosis (Björkbacka et al., 2004; Braga et al., 2012), and blockade of MyD88 increases the severity and mortality of colitis in mice (Rakoff-Nahoum et al., 2004). Altogether, this suggests that MyD88 has different effects in acute versus chronic inflammatory conditions.

We observed that depletion of MyD88 in adipocytes does not protect mice from obesity and IR. In contrast, the absence of MyD88 in myeloid cells improved insulin sensitivity in the HFD-fed mice. These mice had decreased CD11c+ AT macrophage numbers and improved intestinal barrier function. Thus, deletion of MyD88 in myeloid cells improved the AT inflammation and systemic insulin sensitivity. This contrasts the phenotype of our and other reports of the global MyD88 KO mice (Hosoi et al., 2010; Yokoyama et al., 2012) and contrasts the findings indicating that depletion of MyD88 in myeloid cells does not protect mice from obesity-induced IR (Kubinak et al., 2015). However, we cannot exclude the environmental factors at different animal facilities.

Our experiments on depletion of MyD88 in adipocytes and myeloid cells does not exclude the interference of MyD88 expression in other cell types such as epithelial intestinal cells and lymphocytes, which may contribute to the observed metabolic phenotypes in these mice.

Macrophages play an essential role in obesity-induced IR. Here, the HFD-fed LyZMyD88^KO^ mice demonstrate that loss of MyD88 function in myeloid cells prevents CD11c+ AT macrophage polarization, which may account for the improvement in body weight gain, adiposity, glucose homeostasis, and gut permeability. Thus, an MyD88-independent pathway may be involved in the regulation of inflammation-induced metabolic syndrome.

Dectin-1 is involved in fungal infections and was recently reported to play a role in anti-tumor immune responses (Chiba et al., 2014). Moreover, vimentin levels were increased in atherosclerotic plaques and can bind to and activate Dectin-1 in macrophages (Thiagarajan et al., 2013). The role of vimentin in obesity is unknown. We hypothesized that loss of functional MyD88 may induce other factors, such as vimentin in the AT, to induce IR independent of the classical MyD88-dependent inflammatory pathway. We performed proteomics analysis in the AT and found that vimentin was upregulated in the DIO MyD88 KO mice. Therefore, vimentin may in part drive the HFD-induced obesity by directly binding to and activating Dectin-1 (Thiagarajan et al., 2013). Hypoxia is commonly observed in the AT from the HFD-fed mice and has been reported to regulate vimentin expression (Satelli and Li, 2011). Increased vimentin levels may be due to the AT hypoxia in our HFD-induced obesity models. Furthermore, hypoxia was shown to increase the expression of Annexin A1 (ANXA1), which induces prostate cancer cell proliferation (Bizzarro et al., 2016). Our proteomics analysis showed a positive interaction between ANXA and vimentin in the AT of the WT and MyD88 KO DIO mice, suggesting that vimentin is increased during obesity due to low oxygen availability in the AT. Fungal microbiota was undetectable by our ITS sequencing analysis, further validating the role of Dectin-1 and vimentin in obesity-driven inflammation and impaired glucose homeostasis.

During fungal infection, Dectin-1 affects macrophage polarization by activating IRF5 in the Dectin-Syk pathway (del Fresno et al., 2013). IRF5 is a transcription factor that regulates proinflammatory CD11c+ macrophage differentiation (Weiss et al., 2013). We found that the Dectin-1 adaptor molecules Syk and *Irf5* are upregulated in the AT of the obese MyD88 KO mice and demonstrate that Dectin-1 activation contributes to the development of IR. Furthermore, Dectin-1 levels in the CD11c+ AT macrophages were increased in the lean and obese MyD88 KO mice, while the Dectin-1 KO mice were protected from obesity and IR. Blocking Dectin-1 improved insulin sensitivity in the mice fed an HFD and treating the chow- and HFD-fed WT and MyD88KO mice with the Dectin-1-specific agonist worsened glucose homeostasis. Altogether, these data support our idea that even in the absence of MyD88, antagonizing Dectin-1 is sufficient to restore the glucose homeostasis context of MyD88 deletion.

Activation of Dectin-1 increases the TNF-α production in human macrophages (Ferwerda et al., 2008). Also, the Dectin-1 agonist curdlan increases the expression of cell surface markers specific to macrophage and the DCs activation (cluster differentiation [CD]40 and CD86) and increases cytokine release by the DCs (Kim et al., 2016). Moreover, Dectin-1 activation by β-glucan is sufficient to polarize tumor-associated macrophages from a M2 to an M1 phenotype (Liu et al., 2015). These data support our hypothesis that activation of Dectin-1, even in the absence of the global MyD88 in the HFD-fed mice, is sufficient to drive CD11c+ AT macrophage polarization and subsequent onset of IR. Here, the Dectin-1 KO mice also have reduced CD11c+ AT macrophage numbers. Laminarin treatment decreased the proinflammatory profile of the CD11c+ AT macrophages in the lean and DIO MyD88 KO and in WT mice, while blocking Dectin-1 increased anti-inflammatory CD206+ AT macrophage numbers. Conversely, activation of Dectin-1 increased the CD11c+ AT macrophages in all genotypes. Here, we show that Dectin-1 inhibition decreases the number of proinflammatory CD11c+ AT macrophages, which is consistent with Dectin-1-dependent proinflammatory macrophage polarization in tumor and fungal infection models (Liu et al., 2015; Loures et al., 2014).

Our data show that Dectin-1 directly mediates macrophage polarization and subsequently affects insulin sensitivity. These data indicate that Dectin-1 activation is involved in CD11c+ AT macrophages accumulation and inflammation-induced IR.

It is well known that changes in the gut microbiota are involved in the pathogenesis of obesity and IR (Castoldi et al., 2015a; Ley et al., 2005). Obesity is associated with increased *Firmicutes* and decreased *Bacteroidetes* abundance (Ley et al., 2005). We showed that the HFD, but not the absence of MyD88, induces changes on the gut microbiota communities after 90 days of HFD feeding. However, 5 weeks of HFD-feeding induced differences in the gut microbial communities between the WT and MyD88 KO mice. For instance, we found that *Deltaproteobacteria* was increased in both the obese WT and MyD88 KO mice, which is also consistently increased in obesity induced by HFD in our and previous studies (Lecomte et al., 2015). Increased prevalence of *Deltaproteobacteria*, specifically the *Desulfovibrio* genus, is observed in type 2 diabetic patients (Qin et al., 2012) and in patients with persistent diabetes after bariatric surgery (Murphy et al., 2016). This is consistent with our results in obese and insulin resistant mice. Together, our results here show that MyD88 does not control the gut microbial communities after 90 days of HFD-feeding.

The role of Dectin-1 in obesity and its effects on the gut microbiota are unclear. HFD-fed Dectin-1 KO mice showed significant differences in bacterial communities compared to the WT mice in both the lean and DIO states having increased *Bacteroidetes* and decreased *Deltaproteobacteria* abundances. However, laminarin and curdlan were not able to induce changes in the gut microbial communities after 5 weeks of treatment, which may be due to the short treatment period. These data support a role of Dectin-1 activation in regulating insulin sensitivity.

Dectin-1 expression is increased in the AT from the obese individuals and is positively correlated with BMI, CRP, and triglycerides levels. This suggests that Dectin-1 may have therapeutic implications as a biomarker for metabolic dysregulation in humans. Dectin-1 is expressed in peripheral monocytes from type 2 diabetic patients with poor glycemic control (Cortez-Espinosa et al., 2012). Furthermore, the expression of vimentin in the AT from the obese individuals was increased, and we found that Dectin-1 and vimentin directly interact in the human AT. This highlights potential applications for Dectin-1 in the development of therapeutic targets for obesity in humans. However, we recognize that other endogenous ligands may also stimulate Dectin-1 in obese conditions. More research is needed to further elucidate how Dectin-1 is regulated in obesogenic conditions.

In conclusion, we show that Dectin-1 is important for systemic glucose homeostasis by controlling proinflammatory AT macrophage accumulation and by maintaining a healthy gut microbiota profile. We also show that MyD88/Dectin-1 regulation is critical for maintaining glucose homeostasis and systemic inflammation in mice and humans. Collectively, our data provide insight into the roles of MyD88 and Dectin-1 and support a pathway involved in the AT inflammation, which may lead to the development of new therapeutic approaches to treat insulin resistant individuals.

## EXPERIMENTAL PROCEDURES

### Animal Studies

Mouse studies were conducted in accordance with federal guidelines. The Institutional Animal Care and Use Committee (Institute of Biomedical Science, University of Sao Paulo, Sao Paulo, Brazil) approved all studies. Studies were performed on age- and sex-matched littermates. Male mice, 4–8 weeks old, all C57BL/6, MyD88 KO, Dectin-1 KO, and WT. Adiponectin^cre+^ MyD88^flox/flox^ (AdipoMyD88^KO^) mice and Lysozyme^cre+^ MyD88^flox/flox^ (LyZMyD88^KO^) were also used in this study. For more details, see Supplemental Experimental Procedures.

### Human Studies

Mesenteric AT was obtained of six lean and seven obese patients from Hospital Universitário/University of São Paulo, SP, Brazil under the ethics committee number: CEP 1390/14 and CAEE: 20643513.9.000.5467. The individuals were seven males and six females, age range 28–65 years old.

### Obesity Induction

Obesity was induced by the HFD (rodent diet 45% kcal from fat, 20% kcal from protein, and 35% kcal from carbohydrate, Research Diets) irradiated. The mice were fed for 12 weeks starting from the sixth week of life. For more details, see Supplemental Experimental Procedures.

### Metabolic Parameters Analysis

Peripheral response to glucose was assessed by glucose tolerance test (GTT) in mice fasted for 12 hr and injected with 2 g/kg of glucose. The insulin response was examined by insulin tolerance test (ITT) after fasting mice for 6 hr and injection of 0.8 U/kg of insulin. For more details, see Supplemental Experimental Procedures.

### Purification of Infiltrating Cells in AT and Evaluation of Cellular Phenotypes by Flow Cytometry

Cells from the AT were purified and stained with the following antibody panel: anti-CD45, CD11b, F4/80, CD11c, and CD206 diluted 1:100 (BioLegend). M1 macrophages were characterized by expression of CD11c concomitant with F4/80 and CD11b markers and M2 macrophages by expression of CD206 concomitant with F4/80 and CD11b markers. Dectin-1 positive cells were identified using anti-Dectin-1/CLEC7A diluted 1:100 (BioLegend). The characterization of subpopulations of leukocytes was performed on the FACSCANTO II machine (BD), and data analysis was performed with FlowJo 9.5.3 software (Treestar). For more details, see Supplemental Experimental Procedures.

### Gene Expression

After preparation of cDNA, the quantification of gene expression by real-time PCR was performed. Amplification conditions were standardized for each transcript. A comparative relationship between reaction cycles (CT) was used to determine gene expression relative to HPRT control (housekeeping gene). For more details, see Supplemental Experimental Procedures.

### Laminarin and Curdlan Treatment

Laminarin (SIGMA) and Curdlan (SIGMA) diluted in sterile PBS 1 × were administered intraperitoneally (250 mg/kg) and (15 mg/mouse) respectively three times a week during 5 weeks. PBS was administered as vehicle. For more details, see Supplemental Experimental Procedures.

### Analysis of Gut Microbiota Composition

For more details, see Supplemental Experimental Procedures.

### BMDMs

Mouse tibia and femur were harvested and cultivated in DMEM containing 10% fetal bovine serum, 1% penicillin/streptomycin (pen/strep), and 20 ng/mL of M-CSF (PeproTech). After 6 days, the medium was replaced and cells were stimulated with addition of LPS (100 ng/mL), plus IFN-γ (10 ng/mL, PeproTech) for M1 polarization or IL-4 (10 ng/mL, PeproTech), plus IL-13 (10 ng/mL, PeproTech) for M2 polarization for 24 hr. For more details, see Supplemental Experimental Procedures.

### Statistics

All values are given as means ± SEM. Differences among the groups were compared using ANOVA with Tukey post-test for multiple comparisons and Student’s t test when there were only two groups. All statistical analysis were performed using GraphPad PRISM 6 software, and the differences were considered significant when p < 0.05.

## Supplementary Material

1

## Highlights

- MyD88 absence upregulates Dectin-1 in adipose tissue (AT) and AT macrophages (ATMs)
- Dectin-1 inhibition decreases CD11c+ ATMs and protects mice from insulin resistance (IR)
- Dectin-1 activation increases CD11c+ ATMs and worsens IR
- Obese humans have increased Dectin-1 expression in AT
