# Supplementary material for: Dectin-1 Activation Exacerbates Obesity and Insulin Resistance in the Absence of MyD88
Source: Cell Rep. Author manuscript; Available in PMC 2022 Jul 7. (PMC9261359; doi:10.1016/j.celrep.2017.05.059)
Supplement: 1 [file NIHMS1817942-supplement-1.pdf]

Supplemental Tables

Table S1. Comparison of BMI, C reactive protein (CRP), Cholesterol and Triglycerides between lean and obese individuals. Related to Figure 7. Mean BMI values, serum concentration of CRP, cholesterol and triglycerides of lean and obese individuals. P-values are included in the table.

Table S1. Comparison of BMI, C reactive protein (CRP), Cholesterol and Triglycerides between lean and obese individuals

	BMI(Kg/m^2)	CRP(mg/dl)	Cholesterol(mg/dl)	Triglycerides(mg/dl)
**Lean**	23.66	1.557	208.125	153.333
**Obese**	*31.87	*7.3111	$^{\#}$221.222	*221.8333

*<0.05; # p=0.6 vs lean individuals; n=6-7 each group

Table S2. Statistics of unweighted and weighted analysis for lean and DIO, WT and MyD88 KO mice. Related to Figure 1. Comparison between diets (HFD and chow) and genotypes (WT and MyD88 KO) from unweighted and weighted Principal Coordinate (PC) analysis after 90 days of HFD feeding. P-values are indicated. P-values ≤ 0.05 are highlighted in yellow.

Table S2. Statistics of unweighted and weighted analysis for lean and DIO, WT and MyD88 KO mice

			Df	Sums Of Seqs	Mean Sqs	F.Model	R2	p-value
**Lean x DIO**	unweighted	Diet	1	0.67116	0.67116	2.4472	0.25904	0.015
**WT x MyD88 KO**	unweighted	Genotype	1	0.33847	0.33847	1.0518	0.13063	0.284
**Lean x DIO**	weighted	Diet	1	0.74467	0.74467	3.6607	0.34338	0.043
**WT x MyD88 KO**	weighted	Genotype	1	0.42697	0.42697	1.716	0.19688	0.134

Adonis method was used to test strength and statistical significance.

Table S3. Statistics of unweighted and weighted analysis for lean and DIO, WT and Dectin-1 KO mice. Related to Figure 4. Comparison between diets (HFD and chow) and genotypes (WT and Dectin-1 KO) from unweighted and weighted Principal Coordinate (PC) analysis after 90 days of HFD feeding. P-values are indicated. P-values ≤ 0.05 are highlighted in yellow.

Table S3. Statistics of unweighted and weighted analysis for lean and DIO, WT and Dectin-1 KO mice

			Df	Sums Of Seqs	Mean Sqs	F.Model	R2	p-value
**Lean x DIO**	unweighted	Diet	1	0.9698	0.96978	3.3682	0.11091	0.001
**WT x Dectin-1KO**	unweighted	Genotype	1	1.2377	0.61884	2.1436	0.14155	0.001
**Lean x DIO**	weighted	Diet	1	1.0319	1.03186	8.3633	0.2365	0.001
**WT x Dectin-1KO**	weighted	Genotype	1	0.8906	0.44532	3.3343	0.20413	0.001

Adonis method was used to test strength and statistical significance.

Table S4. Statistics of unweighted and weighted analysis for laminarin-and vehicle-treated, WT and MyD88 KO mice. Related to Figure 5. Comparison between diets (HFD and chow), genotypes (WT and MyD88 KO) and treatment (laminarin and PBS) from unweighted and weighted Principal Coordinate (PC) analysis after 5 weeks of HFD feeding. P-values are indicated. P-values ≤ 0.05 are highlighted in yellow.

Table S4. Statistics of unweighted and weighted analysis for laminarin-and vehicle-treated, WT and MyD88 KO mice

			Df	Sums Of Seqs	Mean Sqs	F.Model	R2	p-value
**Lean x DIO**	unweighted	Diet	1	1.4247	1.42466	6.0319	0.17218	0.001
**WT x MyD88KO**	unweighted	Genotype	1	0.5706	0.57063	2.1481	0.06897	0.007
**PBS x Laminarin**	unweighted	Treatment	1	0.3092	0.30924	1.1259	0.03737	0.233
**Lean x DIO**	weighted	Diet	1	1.0339	1.0339	18.856	0.39402	0.001
**WT x MyD88KO**	weighted	Genotype	1	0.23176	0.231762	2.8095	0.08832	0.032
**PBS x Laminarin**	weighted	Treatment	1	0.15093	0.15093	1.7698	0.05752	0.106

Adonis method was used to test strength and statistical significance.

Table S5. Statistics of unweighted and weighted analysis for curdlan- and vehicle-treated, WT and MyD88 KO mice. Related to Figure 6. Comparison between diets (HFD and chow), genotypes (WT and MyD88 KO) and treatment (curdlan and PBS) from unweighted and weighted Principal Coordinate (PC) analysis after 5 weeks of HFD feeding. P-values are indicated. P-values ≤ 0.05 are highlighted in yellow.

Table S5. Statistics of unweighted and weighted analysis for curdlan- and vehicle-treated, WT and MyD88 KO mice

			Df	Sums Of Seqs	Mean Sqs	F.Model	R2	p-value
**Lean x DIO**	unweighted	Diet	1	0.7299	0.72987	1.8713	0.06964	0.001
**WT x MyD88KO**	unweighted	Genotype	1	1.1756	0.58781	1.5161	0.11217	0.001
**PBS x Curdlan**	unweighted	Treatment	1	0.441	0.44102	1.0982	0.04208	0.09
**Lean x DIO**	weighted	Diet	1	0.36592	0.36592	7.9792	0.24195	0.001
**WT x MyD88KO**	weighted	Genotype	1	0.43679	0.218394	4.873	0.2888	0.001
**PBS x Curdlan**	weighted	Treatment	1	0.06168	0.061681	1.0629	0.04078	0.379

Adonis method was used to test strength and statistical significance.

Supplemental experimental procedures

Animal Studies

Mouse studies were conducted in accordance with federal guidelines. The Institutional Animal Care and Use Committee (Institute of Biomedical Science, University of Sao Paulo, Sao Paulo, Brazil) approved all studies. This study was registered under the protocol 110 on the sheet 133 of the book 02/2011, and protocol 122 on the sheet 134 of the book 02/2011. Studies were performed on age- and sex-matched littermates. Male mice, 4-8 weeks old, all C57BL/6, MyD88 knockout (KO) and normal Wild Type (WT) were used. Adiponectin^{cre} MyD88$^{flox/flox}$ (AdipoMyD88^{KO} and AdipoMyD88^{WT}) mice and Lysozyme^{cre} MyD88 $^{flox/flox}$ (LyZMyD88^{KO} and LyZMyD88^{WT}) were also used in this study. C57BL/6 and MyD88 KO mice were provided by the vivarium of inbred mice at the University of Sao Paulo and CEDEME at the Federal University of Sao Paulo. MyD88$^{flox/flox}$ mice (number 008 888; B6.129P2 (SJL) -Myd88tm1Defr/J) were purchased from "The Jackson Laboratory" and Adiponectin^{cre+} mice (number 010 803; B6.FVB-Tg (ADIPOQ-cre) 1Evdr J) and Lysozyme^{cre+} (number 004781; B6.129P2-Lyz2tm1 (Cre) Ifo/J) in order to generate the Adiponectin^{cre+} mice MyD88$^{flox/flox}$ and Lisozyme^{cre+} MyD88$^{flox/flox}$ were provided by Professor Dr. Marcelo Mori from Department of Biochemistry and Tissue Biology of University of Campinas-Brazil, and Professor Dr. William T. Festuccia from Department of Physiology and Biophysics of University of Sao Paulo-Brazil, respectively. Dectin-1 KO mice were provided by Dr. Gordon D. Brown from MRC Centre for Medical Mycology, Aberdeen Fungal Group, School of Medicine, Medical Sciences & Nutrition, Institute of Medical Sciences, University of Aberdeen, Aberdeen, United Kingdom. The mice were bred and genotyped in our animal facility. During the diet, animals were placed in collective microisolators containing up to five animals, with artificial light-dark cycle of 12 hours, at a constant room temperature of 22 °C, and water supplies and irradiated food available at all the time. For studies with MyD88 KO mice, WT mice were used as controls from the same vivarium and matched for age and sex. For experiments with mice Adiponectin^{cre+} mice MyD88$^{flox/flox}$ (AdipoMyD88^{KO}), Adiponectin^{cre-} mice MyD88$^{flox/flox}$ (AdipoMyD88^{WT}) mice were used as control. For experiments using Lysozyme^{cre+} MyD88$^{flox/flox}$ (LyZMyD88^{KO}) mice, Lysozyme^{cre-} MyD88 $^{flox/flox}$ (LyZMyD88^{WT}) mice were used as control. For all groups fed with irradiated high fat diet, the same number of mice was fed with standard irradiated diet.

Human Studies

Mesenteric adipose tissue was obtained of six lean and seven obese patients from Hospital Universitário/University of São Paulo, SP, Brazil under the ethics committee number: CEP 1390/14 and CAEE: 20643513.9.000.5467. The individuals were seven males and six females, age range 28-65 years-old.

Obesity Induction

Obesity was induced by irradiated high fat diet (Rodent Diet 45% kcal from fat, 20% kcal from protein and 35% kcal from carbohydrate, Research Diets, Inc.). The mice were fed for 12 weeks starting from the 6th week of life. The normal (chow) diet used as control contains, by its turn, 24% protein, 47.5% carbohydrate, and 4.9% fat. Mice weight and food intake were monitored constantly.

Metabolic Parameters Analysis

Peripheral response to glucose was assessed by glucose tolerance test (GTT). Glucose (2g/ Kg body weight) was administered intraperitoneally in mice fasted for 12 hours. Glucose levels in blood were determined before and after 15, 30, 60, 90 and 120 minutes from glucose administration. The insulin response (insulin tolerance test (ITT) was examined after fasting mice for 6 hours. Blood glucose levels were determined before and after 15, 30, 60, 90 and 120 minutes of 0.8 U/kg insulin administration. All mice were maintained in individual cages. It was used ACCU-CHEK®Advantage II - (Roche Mannheim, Germany) for reading the blood glucose levels.

Body Fat Evaluation by DEXA

Imaging of lean and obese mice was performed in the imaging *in vivo* system Carestream MS FX multispectral PRO. X-ray images were taken at different power levels: a low energy X-ray for images of soft tissues (fat), and a high energy X-ray for images of bone. The images were automatically corrected for illumination, and tissue density of each mouse was obtained. The overlapping photos with different energy levels enabled us to differentiate fat, lean and bone mass. The calculation performed to obtain the percentage of total fat is described below:

$$\% \text{ Fat} = \frac{(\text{Area [fat + lean mass + bone mass] -Area [lean mass + bone mass]}) \times 100}{\text{Area [fat + lean mass + bone mass]}}$$

The experiment and analysis were performed at the Research Support Facilities Center (CEFAP) at University of Sao Paulo-Brazil.

Oxygen Consumption/Carbon Dioxide Production and Determination of Respiratory Exchange Ratio (RER)

Oxygen consumption/carbon dioxide production, respiratory exchange ratio (RER), and activity were measured in animals fed through an indirect open circuit calorimeter (Oxymax system luxury; Columbus Instruments, Columbus,

Ohio). Oxygen consumption was measured after a 24 hour induction period in metabolic cages. The animals were kept at 24-hour rest in individual metabolic cages specific for measurements of small animals at rest. The system was calibrated with a mixture of gases consisting of 20.5% oxygen and 0.5% carbon dioxide. Oxygen uptake (mL.kg -1 .min -1), carbon dioxide production (mL.kg -1 .min -1), and respiratory exchange ratio (RER) were then measured.

Serum Insulin Quantification
Blood was collected after fasting for 12 hours and after 30 and 90 minutes of injection of 2g/kg glucose. Serum free of hemolysis was generated by centrifugation. Ten microliters of serum were used for the assay. Insulin was measured by an ELISA kit from Millipore (Billerica, MA, USA), the kit sensitivity is 0.2 ng/mL. Samples were quantified in duplicates. The measurement was performed according to manufacturer's recommendations.

Determination of Serum LPS
LPS concentration in serum was determined using a chromogenic assay based on a Limulus amebocyte extract (LAL kit-terminal QCL1000) (LONZA, Basel, Switzerland). Samples were collected at the time of euthanasia via cardiac puncture to try to minimize as much as possible the chance of contamination. The test is quantitative for Gram-negative bacterial endotoxin and was carried out according to manufacturer's guidelines. We used 50 microliters of sample in duplicates. The kit's sensitivity is 0.1 endotoxin units/mL.

Proteomics Analysis
In-solution tryptic digestion: All protein solutions were previously quantified using Qubit® (fluorometric quantitation) Protein Assay Kit. From those solutions, an equivalent aliquot of 50 µg of protein was transferred to 0.5 ml tubes and dried down. The protein pellets were resuspended in 25 µl of Urea 6 M. 25 µl of a reducing reagent, DTT 10 mM was added, and the samples were reduced for 60 min at room temperature. Subsequently, 50 µl of an alkylation solution, IAA 100 mM, was added, and the samples were alkylated for another 60 min at 54 °C in the dark. Afterwards, 1 µl of DTT 1M was added to react with the remaining IAA. Finally, 100 µl of ice-cold trypsin solution at 1:50 (trypsin/protein) ratio was added to the samples, followed by incubation for 16 h at 37 °C. Following digestion, the reaction was stopped by adding 5 µl of formic acid 10%. Finally, the samples were desalted using ZipTips® and kept at -20 °C until the analysis.
LC-MS/MS Analysis: Peptides were analyzed by on-line nanoflow LC-MS on an EASY-nLC II system (Thermo Scientific) connected to an LTQ-Orbitrap Velos instrument (Thermo Scientific) via a Proxeon nanoelectrospray ion source. Peptides were separated on an analytical EASY-Column (10cm, ID75µm, 3µm, C18-Thermo Scientific) previously trapped in a pre-column EASY-Column (2cm, ID100µm, 5µm, C18-Thermo Scientific). Tryptic digested peptides were separated using a 60-min linear gradient of 0–60% buffer B (acetonitrile in 0.1% formic acid) at 300nL/min flowrate. The LTQ-Orbitrap Velos mass spectrometer was operated in positive ion mode using DDA (data-dependent acquisition) mode, in which the 20th most intense precursor ions from a full MS scan were selected for fragmentation by CID (collision induced dissociation). Full MS scans were performed with 60,000 of resolution, and the m/z range for MS scans was 400–1200. The normalized CID collision energy was 35 eV for a doubly charged precursor ion with isolation with of 2 m/z, activation Q of 0,250 and activation time of 10 ms. The minimum signal threshold was 15000 counts and, for dynamic exclusion, it was considered 1 repeat count with a duration of 30 s. In order to discriminate the charge state of the peptides, the charge state screening was enabled, and ions either with unassigned charge state or singly charged were rejected.
Database search: The MS/MS spectra from each LC-MS/MS run were searched against five different databases with two different search engines an in-house Proteome Discoverer 1.4 software (Thermo, USA). The search criteria were as follows: full tryptic specificity was required, two missed cleavage was allowed, carbamidomethylation (C) was set as the fixed modification, and the oxidation (M) was set as the variable modification, precursor ion mass tolerances were set at 10 ppm for all MS acquired in an orbitrap mass analyzer, and the fragment ion mass tolerance was set at 0,6 Da for all MS2 spectra acquired. All covariates were log-transformed before statistical analysis. All the analyses were performed using STRING software and UniProt for protein-protein interactions, identification and statistics. $P \leq 0.05$ were considered significant.

Determination of Intestinal Permeability by FITC-DEXTRAN
Intestinal permeability was evaluated by gavage of FITC–DEXTRAN (FITC-DX-4000) (Sigma, St. Lous, MO, USA), a macromolecule which is not metabolized, and thus used as a permeability probe. After 12 weeks of high fat diet, 5 mice from each group were treated by gavage with FITC-dextran (250 mg / kg) after 4 hours of fasting. After 6 hours, blood was collected from the tail. FITC-dextran dilutions in PBS were used as a standard curve (0, 1.25, 2.50, 5.0, 10, 20, 40, 60, 80 ug/ml) and fluorescence of 100µL of serum or standard was measured in a fluorometer with excitation at 485 nm and reading at 535 nm. Serum from a mouse without FITC-dextran administration was used as negative control.

Triglycerides and Cholesterol Measurement
Mice were fasted for 12 hours and blood was collected from the tail. Hemolysis free serum was generated after centrifugation. Cholesterol was analyzed by enzymatic colorimetric test BIOCLIN (Cholesterol monoreagent K083, BIOCLIN, Belo Horizonte, MG, Brazil), and triglycerides by colorimetric assay (Triglyceride liqueform, LABTEST, MG, Brazil) as recommended by manufacturers.

AT Carbonyl Content Quantification
Oxidative damage to proteins was determined by the carbonyl levels in total delipidated AT homogenate. Samples were incubated with 20 mM 2,4-dinitrophenylhidrazine in 2 N HCl for 1 hour at 37°C. Afterward, proteins were precipitated with 12% TCA (final concentration) and centrifuged for 10 minutes at 5000xg. Pellet was washed with cold ethanol: ethyl acetate (1:1) until organic phase appeared optically clear. Excess solvent was eliminated by washing the protein pellet with phosphate buffer (K_2HPO_4 20 mM, pH 6.8), and samples were again precipitated with 12%TCA. Pellet was dissolved in 6N guanidine hydrochloride. Carbonylated proteins were quantified spectrophotometrically at 460-470 nm using a molar extinction for hydrazine derivatives of 22,000 M^{-1} cm^{-1} (Levine et al., 1990).

Purification of Infiltrating Cells in Adipose Tissue and Evaluation of Cellular Phenotypes Flow Cytometry
For purification of fat and liver tissues infiltrating cells, the tissue was incubated with type II collagenase (10 mg/ml; Sigma-Aldrich) in PBS with EDTA (2 mM; Sigma-Aldrich) for 30 minutes at 37 °C. The AT suspension was filtered using a cell strainer 100 micrometers. The solution was subjected to Percoll gradient for purification of leukocytes. The liver was digested in type IV collagenase (2 mg/ml; Sigma-Aldrich) in PBS with EDTA (2 mM) for 30 minutes at 37 °C. The tissue suspension was filtered using a cell strainer 100 micrometers. The solution was subjected to Percoll gradient for purification of leukocytes. After Percoll gradients, cells were washed with PBS and stained with the following antibody panel: anti-CD45, CD11b, F4/80, CD11c, CD206 and CD86
diluted 1:100 (BioLegend, CA, USA). Proinflammatory macrophages were characterized by expression of CD11c concomitant with F4/80 and CD11b markers, and anti-inflammatory macrophages by expression of CD206 concomitant with F4/80 and CD11b markers. Dectin-1 positive cells were identified using anti-Dectin-1/CLEC7A diluted 1:100 (Biolegend, CA, USA). The characterization of subpopulations of leukocytes was performed on the FACS CANTOII machine (BD, Beckton Dickson, NJ, USA) and data analysis was performed with FlowJo 9.5.3 software (Treestar).

Western Blotting
Approximately 50 μg of total protein, obtained from epididymal AT or intestine, were diluted in sample buffer (Biorad, USA) containing 20 mg/mL of 2-β-mercaptoethanol (Sigma-Aldrich). Proteins were denatured by heating for 5 minutes at 95°C, and were then separated by electrophoresis on a 10% polyacrylamide gel. Subsequently, the proteins were transferred to a nitrocellulose membrane, and blocked for 1 hour with 5% milk dissolved in TBS-Tween, and then incubated with the primary antibody diluted in TBS-Tween. Next, the membrane was washed with TBS-Tween and incubated for 1 hour with the biotinylated secondary antibody. The molecular mass of protein was determined by comparison with the migration pattern of Rainbow protein or Dual Color (Biorad Laboratories, CA, USA). The primary antibodies used were mouse p-JNK, p-IκB-α mouse, p-AKT 473, p-AKT 308 Total AKT, Claudin-1 (1: 1000 Cell Signaling Technology, MA, USA), MyD88 (Santa Cruz SC-11356), α- tubulin (SC-5286) and β-actin (Sigma-Aldrich, 1: 10,000), Vimentin (Sigma V2258) and Dectin-1/CLEC7A (Santa Cruz SC-26094).

Co-Immunoprecipitation
Approximately 800 μg of total protein, obtained from human AT, were co-immunoprecipited using Pierce Co-Immunoprecipitation (Co-IP) Kit 26149 (Pierce Biotechnology, IL, USA), following manufacturer instructions. Purified anti-human Dectin-1 (CLEC7A) antibody (Biolegend 355402) and anti-IgG (Southern Biotech, 2040-08) were used for antibody immobilization and to pull down Dectin-1 from AT. Anti-Vimentin from Sigma (V2258) and anti-Dectin-1/CLEC7A from Santa Cruz (SC26094) were used to detect the interaction between Vimentin and Dectin-1.

RNA Extraction and cDNA Preparation
Samples of epididymal AT, intestine fragments, and BMDM were submitted to RNA extraction using Trizol reagent (Life Technologies, CA, USA). Preparation of complementary DNA (cDNA) was performed using 2 ug of RNA, 0.8 μL of reverse transcriptase M-MLV, 4 uL of 5X Reaction Buffer M-MuLV, 2 uL of 10 mM dNTPs, 32 U/uL of RiboLock RibonucleaseInhibitor ™ (all from Fermentas, Hanover, MD, USA) and 320 ng of oligo-dT primer (Integrated DNA Technologies, Coralville, IA, USA). This mixture was incubated at 42 ° C for 60 minutes, and then at 70 ° C for 10 minutes. cDNA was stored at -20 ° C.

Real Time PCR
After preparation of cDNA, the quantification of gene expression by real-time PCR was performed. Amplification conditions were standardized for each transcript. A comparative relationship between reaction cycles (CT) was used to determine gene expression relative to HPRT control (housekeeping gene). The detection of the gene of interest was performed using ABI Prism 7300 apparatus (Applied Biosystems). Commercially available probes (Life Technologies) for the following genes were used: *Il-1β* (Mm01336189_m1), *Il-6* (Mm_00561420_m1), *Tnf-α* (Mm_00443258_m1) and *Hprt* (Mm03024075_m1). The following sequences of primers, detected using SybrGreen® (Life Technologies) were also used for this study: *RegIII-γ* forward: TTCCTGTCCTCCATGATCAAAA and reverse: CATCCACCTCTGTTGGGTTCA, *Defcr-1* forward: CAGGCCGTATCTGTCTCCTT and reverse: ATGACCCTTTCTGCAGGTTC, *Zo-1* forward: AGGACACCAAAGCATGTGAG and reverse: GGCATTCCTGCTGGTTACA and KiCqStart Primers Human (H)_*Arg-1* (NM_001244438), H_*Il-6* (NM_000600), H_*Irf5* (NM_001098627), H_*Clec7a* (Dectin-1) (NM_022570), H_*Syk* (NM_001135052), Mouse (M)_*Clec7a* (Dectin-1) (NM_020008), M_*Irf5* (NM_012057), M_*Syk* (NM_011518) and M_*Arg-1* (NM_007482) were purchased from

Sigma Aldrich. The quantification method was 2-ΔΔCt (Livak and Schmittgen, 2001) using control samples from lean mice or lean human as a normalizer.

CBA (Cytometric Bead Array)

The kit Mouse Cytokine CBA Assay (BD Bioscience) was used to quantify TNF-α and IL-6 in epididymal AT and serum. The test was carried out according to manufacturer's directions. The samples were acquired on FACS CANTO II device (BD Biosciences), and analyzed using FCAP Array software (BD Biosciences).

Analysis of Glucose Incorporation and Conversion to Its Metabolites in vitro

To study the *ex vivo* cell metabolism, we used AT fragments, instead of isolated adipocytes, to avoid metabolic stress associated with collagenase digestion, and to preserve the microenvironment. The interpretation of data should therefore take into account the possibility that other cell populations that compose AT, as well as mature adipocytes, may have contributed to the observed effects. Briefly, epididymal AT fragments (20-25 mg) were incubated in 1 ml Krebs bicarbonate buffer -Ringer (mmol/L): 118 NaCl, 4.8 KCl, 1.25 $CaCl_2$, 1.2 KH_2PO_4, 1.2 $MgSO_4$, 25 $NaHCO_3$, and 5.5 [U-14C] glucose (0.5 mCi/tube 3mCi/mmol, Amersham) supplemented with 2.5% fatty acid free BSA (Sigma - Aldrich, Oakville, ON, Canada), pH 7.4. The vials were closed with rubber stoppers, gassed with 5% CO_2 and 95% O_2, and incubated at 37 °C for 1 hour, in the presence or absence of insulin (100 pmol / L). At the end of the incubation, the reactions were stopped with H_2SO_4, and benzethonium hydroxide was injected with a syringe and needle in a previously positioned strip of paper on the rubber stopper to hold CO_2. The fragments were removed and intended for extraction of lipids, and hydrolysis of TAG. Afterwards, radioactivity was detected by a beta counter. Values were expressed in DPM (disintegrations per minute) and normalized by tissue weight.

Laminarin Treatment

Laminarin (Sigma), diluted in sterile PBS1X, was administered intraperitoneally (250 mg/Kg), 3 times a week, during 5 weeks, in mice fed chow and/or HFD.

Curdlan Treatment

Curdlan (Sigma), diluted in sterile PBS1X, was administered intraperitoneally (15 mg/mouse), 3 times a week, during 5 weeks. PBS was administered as vehicle in mice fed chow and/or HFD.

Analysis of Gut Microbiota Composition

Fecal samples were collected in metabolic cages, frozen in liquid nitrogen and kept at -80°C until use. The DNA was then extracted using the QIAamp DNA Mini Stoolkit (Qiagen, Hilden, Germany) and quantified. 16S amplicon sequencing was performed at CEFAP/ICB-USP. The 16S rRNA gene was amplified using primer pairs selected from Klindeworth, 2013 (Klindworth et al., 2013) targeting the V3 and V4 hypervariable region. Amplicons were generated according to the Illumina MiSeq 16S Metagenomic Sequencing Library Preparation protocol and multiplexed using the Nextera XT Index kit. The quality of libraries was assessed using Bioanalyzer (Agilent), and quantified using Qubit (Life Technologies). Prepared libraries were pooled and then sequenced in a paired-end 2x300bp format on an Illumina MiSeq platform. Quantitative Insights Into Microbial Ecology (Qiime) analysis was done by Tau GC Bioinformatics. Paired-end sequence experiments had read pairs joined with PEAR (v0.9.10) using default settings (p:0.01,v:10,m:0,n:50,t:1,q:0,u:1,g:1,e,s:2,b:33,c:40). Sequence analysis was done using the Qiime package version 1.9.1 and enclosed reference Greengenes database from August 2013. OTU picking and taxonomic assignment used pick_de_novo_otus.py script with default parameters, i.e.: 97% sequence identity, first (seed) representative sequence and consensus taxonomy assigned using uclust (v1.2.22q); alignment with PyNAST (v1.2.2), remove all gaps positions and mask non-conserved positions (Lane, D.J. 1991); representative OTU sequence tree building with FastTree (v2.1.3). OTU networks were generated using Qiime´s make_otu_network.py script and graphed using Cytoscape 3.4 with Allegro Edge-Repulsive Fruchterman-Reingold Layout. Taxonomy summaries figures were done without count normalization (collapse_samples.py). Alpha diversity was done with rarefaction (alpha_rarefaction.py) using 10 steps of both the median number of sequences between samples and 5000 sequences. Beta diversity was calculated with jackknifing for both weighted and unweighted UniFrac (v1.5.3) (jackknifed_beta_diversity.py). Sequence sampling depths used was 17000 with 10 iterations. The jackknife consensus trees were plotted with samples in common between iterations and support values for nodes. Also, a principal coordinate analysis was constructed and plotted (Emperor v0.9.51) based on the rarefied UniFrac distance matrices. Adonis method was used to test strength and statistical significance of sample groupings on each category (Diet, Genotype, Treatment) of the experimental designs (compare_categories.py). The unrarefied beta diversity UniFrac distance matrices were used. Both Lactobacillus murinus and Desulfovibrionaceae species were submitted to a finer grained analysis. Reference sets of sequences for each group of species were compiled from the Silva Database (www.arb-silva.de) on february 2017. Both the genus´ and family´s sequences, as designated by the initial taxonomic analysis, were retrieved and submitted to taxonomic designation (assign_taxonomy.py) using the Silva reference sets.

ITS sequencing was performed by Neoprospecta Microbiome Technologies. ITS region was amplified using the ITS1 (GAACCWGCGGARGGATCA) and ITS2 (GCTGCGTTCTTCATCGATGC) primers, with Illumina adapters, necessary for sequencing. The sequencing was performed in Illumina MiSeq, using V2 kit, with a single-end 300nt run. The bioinformatics analysis for ITS sequencing was performed by Neoprospecta Microbiome Technologies. The primers

and adapters sequences were trimmed from the reads, and only sequences with 275nt or more were used in downstream analysis. Then, all reads with one or more indeterminate bases “N” and truncated sequences with two or more consecutive bases with quality scores below to Q30, were eliminated. OTU Picking was performed using Blast 2.2.28 against UNITE database. To attribute taxonomy, only sequences with hits of 99% of identity in an alignment covered over 99%, were considered.

Bone Marrow-Derived Macrophages (BMDM)
Mouse tibia and femur were harvested, cleaned from adherent tissue, immersed in 70% ethanol for 4 min, and transferred to sterile PBS. Bone extremities were cut, and the marrows were flushed with PBS, centrifuged at 1500 rpm, 4 °C, and incubated for 5 min with red blood cell lysis buffer. Cells were centrifuged, resuspended in DMEM containing 10% fetal bovine serum, 1% pen/strep and 20ng/mL of M-CSF (Peprotech, Rock Hill, NJ, USA), filtered and plated at density of 0.5×10^6 cells per well in six-well plates. Medium was changed after 3 days. After six days, medium was replaced and cells were stimulated with addition of LPS (100 ng/mL) plus interferon (IFN)-γ (10 ng/mL, Peprotech) for M1 (showed as F4/80+ CD86+) polarization, or IL-4 (10 ng/mL, Peprotech) plus IL-13 (10 ng/mL, Peprotech) for M2 (showed as F4/80+CD206+) polarization for 24 h. For analysis of MyD88 expression in BMDM from LyZMyD88^{KO} or LyZMyD88^{WT} and AdipoMyD88^{KO} or AdipoMyD88^{WT}, cells were harvested after 7 days of culture, without any stimulation.

Isolation of Mature Adipocytes and Stromal-Vascular Fraction
Epididymal adipose tissue was digested with collagenase type II (1 mg/mL, Sigma-Aldrich) in Krebs–Hepes buffer containing bovine serum albumin (BSA, 1%) and glucose (2 mmol/L), pH 7.4 at 37°C. After filtering, cell suspension was centrifuged (600 g, 10 min) for isolation of floating mature adipocytes and stromal-vascular cells (pellet) for western blotting.

Co-Culture of Adipocytes and M1 Differentiated BMDM Medium
3T3-L1 cells were cultivated in 6 well plates with DMEM; medium was changed every 2-3 days. When cells were 70% confluent, media was changed to DMEM-low glucose 15% FBS, dexamethasone 1 µM , IBMX 0.5 mM, insulin 10 µM, indomethacin 100 µM and rosiglitazone 5 µM (Sigma, EUA). After 15 days, the differentiated adipocytes were washed and co-cultured with 50% of medium from M0 and M1 macrophages cultures during 24 hours. After 24 hours, medium was replaced, and adipocytes were stimulated with insulin 100nM during 5 min, and then cells were washed and collected for western blotting analysis.

Mice Genotyping
Adiponectin^{cre+}, MyD88$^{flox/flox}$ and Lysozyme^{cre+} mice were crossed for 3 generations until we get the Adiponectin^{cre+} MyD88$^{flox/flox}$ (AdipoMyD88^{KO}) mice and Lysozyme^{cre+} MyD88$^{flox/flox}$ (LyZMyD88^{KO}). First, Cre^{+} mice were crossed with Flox$^{+/+}$ mice (F1) to generate Cre$^{+/-}$ flox$^{+/-}$. After, cre$^{+/-}$ flox$^{+/-}$ mice were bred with Cre$^{+/-}$ flox$^{+/-}$ (F2) to obtain Cre$^{+/+}$ flox$^{+/+}$ mice. To expand the number of animals, Cre$^{+/+}$ flox$^{+/+}$ mice were bred with Cre$^{-/-}$ flox$^{+/+}$ mice (F3) to generate Cre^{+} flox$^{+/+}$ mice and Cre^{-} flox$^{+/+}$ mice. Before the experiments, all mice were genotyped by PCR using DNA isolated from a small piece of tail, and the floxing efficiency was measured by western blotting.

Statistics
All values are given as means±SEM. Differences among groups were compared using ANOVA with Tukey post-test for multiple comparisons, and Student’s t test when there were only two groups. All statistical analyses were performed using GraphPad PRISM 6 software, and the differences were considered significant when $p<0.05$.

References

Klindworth, A., Pruesse, E., Schweer, T., Peplies, J., Quast, C., Horn, M., and Glöckner, F.O. (2013). Evaluation of general 16S ribosomal RNA gene PCR primers for classical and next-generation sequencing-based diversity studies. Nucleic Acids Res 41, e1.

Levine, R.L., Garland, D., Oliver, C.N., Amici, A., Climent, I., Lenz, A.G., Ahn, B.W., Shaltiel, S., and Stadtman, E.R. (1990). Determination of carbonyl content in oxidatively modified proteins. Methods Enzymol 186, 464-478.

Livak, K.J., and Schmittgen, T.D. (2001). Analysis of relative gene expression data using real-time quantitative PCR and the 2(-Delta Delta C(T)) Method. Methods 25, 402-408.
